# Essential and Toxic Elements in Infant Cereal in Brazil: Exposure Risk Assessment

**DOI:** 10.3390/ijerph21040381

**Published:** 2024-03-22

**Authors:** Michele C. Toledo, Janice S. Lee, Bruno Lemos Batista, Kelly P. K. Olympio, Adelaide C. Nardocci

**Affiliations:** 1 School of Public Health, University of São Paulo, São Paulo 01246-904, SP, Brazil; kellypko@usp.br (K.P.K.O.); nardocci@usp.br (A.C.N.); 2 United States Environmental Protection Agency, Research Triangle Park, NC 27711, USA; lee.janices@epa.gov; 3 Center for Natural and Human Sciences, Federal University of the ABC, Santo André 09210-170, SP, Brazil; bruno.lemos@ufabc.edu.br

**Keywords:** rice, metals, early exposure, food safety

## Abstract

Infant cereals, one of the first solid foods introduced to infants, have been reported to pose risks to human health because they contain toxic elements and an excess of essential elements. The objective of this study was to assess the cancer and non-cancer risk of exposure to essential and toxic elements in infant cereal in Brazil. In our analyses, we included data from 18 samples of infant cereals made from different raw materials and estimated the incremental lifetime cancer risks and non-cancer hazard quotients (HQs) for their consumption. Rice cereal is particularly concerning because it is immensely popular and usually contains high levels of inorganic arsenic. In addition to arsenic, we assessed aluminum, boron, barium, cadmium, chromium, copper, lead, manganese, nickel, selenium, silver, strontium, and zinc. The cancer risk was highest for rice cereal, which was also found to have an HQ > 1 for most of the tested elements. Inorganic As was the element associated with the highest cancer risk in infant cereal. All of the infant cereals included in this research contained at least one element with an HQ > 1. The essential and non-essential elements that presented HQ > 1 more frequently were zinc and cadmium, respectively. The cancer and non-cancer risks could potentially be decreased by reducing the amount of toxic and essential elements (when in excess), and public policies could have a positive influence on risk management in this complex scenario.

## 1. Introduction

Infant cereals are one of the first solid foods introduced to infants [1]. They can be classified as processed cereal-based foods, prepared primarily from one or more milled cereals, starchy root products, or both. In general, they are simple cereals and must be reconstituted with milk or another appropriate nutritious liquid [2]. There is concern about the health risks related to excesses of essential elements and the presence of toxic elements in some types of infant cereals [3,4].

Rice cereals are notable for typically having high concentrations of arsenic (As), which are associated with some cancer and non-cancer effects [5,6,7]. Rice is known to contain higher levels of As than other crops, which makes rice and rice products significant sources of human exposure to As [6,8]. Because of their broad availability, bland taste, nutritional value, and relatively low allergic potential, rice cereals are widely used during the weaning and feeding of young children. Consumption is particularly high for infants and young children affected by celiac disease, as rice is gluten-free [4,9]. It has been suggested that the consumption of infant cereal in Brazil is a major source of exposure to some essential and non-essential elements [10].

In addition to the health risks posed by As, the fact that infant cereals made from different raw materials contain other non-essential elements has been raised as a potential cause for concern [11,12]. For example, Hernández-Martínez and Navarro-Blasco [12] reported levels of lead (Pb) and cadmium (Cd) in infant cereals. Exposure to inorganic As (iAs) is associated with some cancer and non-cancer effects [5,13]. The International Agency for Research on Cancer (IARC) classifies iAs as a group 1 carcinogen based on sufficient human evidence that iAs causes skin, lung, and bladder cancer [14], and the United States Environmental Protection Agency (U.S. EPA) classifies iAs as Group A, a human carcinogen [7]. Like As, cadmium (Cd) and lead (Pb) are known to be toxic and carcinogenic [15,16,17,18]. According to the World Health Organization (WHO), As, Cd, and Pb are on the list of the 10 chemicals of greatest public health concern [19]. 

Age at exposure also plays an important role in this scenario, especially regarding exposure to metals. Infants and children have high intestinal absorption capability, which can increase the health risk associated with such exposure [20]. Children also have metabolic processes and a detoxification system that are still developing, as well as presenting higher food consumption by body weight (BW) compared with adults, which makes exposure to hazardous chemicals through diet an issue [4]. The characteristics of the exposed individuals, such as sex, genetics, and nutritional status, are also important [16]. Early exposure to Pb is associated with damage to the neurological system, which can manifest as attention deficit, difficulty concentrating/learning, decreased motor skills, or increased aggressive behavior [21,22,23,24].

Other grains used in the processing of infant cereals include wheat, corn, cornstarch, and oat flour. Because such cereals are usually enriched with specific nutrients, they have become a good option as a complimentary food for infants and children [1,2,25]. Fortifying foods with essential elements usually has a positive effect on public health [25,26]. However, in some cases, fortification can increase the risk of adverse health effects, such as those associated with the consumption of copper (Cu), manganese (Mn), and zinc (Zn), as described by Garcia-Casal et al. [25].

Quantitative risk assessment has been recognized by the FAO as an important tool for risk management and decision-making in public health [27]. A noteworthy study in this regard was conducted by Shibata et al., who performed a risk assessment for rice cereal, considering the presence of arsenic and its consumption by children [4].

The objective of this study was to determine whether the levels of As, as well as those of other essential and toxic elements, in infant cereal consumed by infants and toddlers between 4 and 24 months of age in Brazil pose a risk of deleterious effects to health and to potentially inform appropriate risk management measures. To that end, we evaluated the cancer and non-cancer risks of the consumption of rice cereal and non-rice-based cereal in Brazil.

## 2. Materials and Methods

This study adhered to a risk assessment framework, which involved hazard identification (described in the Section 1), dose–response assessment (slope factor, reference dose, and minimal risk level are presented throughout this section), exposure assessment (average daily dose and incremental lifetime cancer risk), and risk characterization (presented in the Section 3). A deterministic assessment, rather than a probabilistic one, was adopted due to limited data availability.

### 2.1. Average Daily Dose, Carcinogenic Risk, and Hazard Quotient

Assuming that exposure to the elements present in infant cereal occurs between 4 and 24 months of age, we estimated the average ingested daily dose as follows [28]:

(1) $${ADD}_{j}=\frac{\left[C\times {IR}_{j}\times {ED}_{j}\times {EF}_{j}\right]}{\left[BWj\times AT\right]}\;$$

where *ADD_j_* is the average daily dose (mg/kg per day) for the age group *j* (4 to <12 months and 12 to <24 months), *C* is the chemical concentration (ng/g) in an infant cereal, *IR_j_* is the ingestion rate of infant cereal (g/day) for the age group *j*, *ED_j_* is the duration of exposure (8 or 12 months, which are 0.67 and 1 year, respectively) for the age group *j*, *EF_j_* is the exposure frequency (days/year) for the age group *j*, *BW_j_* is the body weight (kg) for the age group *j*, and *AT* is the average time (days).

The cancer risk was estimated for each age group *j*, and the incremental lifetime cancer risk (ILCR) for each infant cereal was calculated by summing the risk related to each age group according to the following equation [29]:

(2) $$ILCR=\sum_{j=1,n}^{\;\;}({ADD}_{j}\times SF)\times \frac{{ED}_{j}}{LT})$$

where *SF* is the slope factor ((mg/kg·day)^−1^) for the carcinogenic element, *LT* is the lifetime (70 years), and *n* is the number of age intervals.

The non-cancer effects were assessed by estimating the fractional hazard quotient for each age group and each element. After summing those, we obtained the overall hazard quotient (HQ) with the following equation:

(3) $$HQ=\sum_{j=1,n\;}^{\;\;}(\frac{{ADD}_{j}}{RfD})\;\times \frac{{ED}_{j}}{ED}$$

where *RfD* is the reference dose (mg/kg·day). The fractional HQ was weighted for the *ED_j_* in relation to the total duration of exposure from 4 months to 24 months of age (1.67 years). The parameter values adopted for the risk assessment are described in Table 1.

### 2.2. Concentrations of Elements in Infant Cereals

Data regarding the concentrations of essential and non-essential elements in infant cereals were obtained from the study conducted by Pedron et al. [10], one of the few works investigating the concentrations of essential and non-essential elements in infant cereal in Brazil to which we had access. The authors obtained 18 samples of eight different brands of infant cereal, available in grocery stores all over the country: rice cereal (*n* = 9), multi-grain cereal containing rice (*n* = 5), and non-rice-based cereal (*n* = 4). The samples were acquired in the 2014–2015 period from different markets in three Brazilian states (São Paulo, Rio Grande do Sul, and Minas Gerais) and the Federal District of Brasília.

The total concentrations of 22 essential and non-essential elements—silver (Ag), aluminum (Al), As, boron (B), barium (Ba), bismuth (Bi), calcium (Ca), Cd, cobalt (Co), Cr, Cu, iron (Fe), potassium (K), lithium (Li), magnesium (Mg), Mn, nickel (Ni), sodium (Na), Pb, selenium (Se), strontium (Sr), and Zn—were determined by inductively coupled plasma mass spectrometry. Arsenic speciation was conducted by using a high-performance liquid chromatograph (HPLC) coupled to the mass spectrometer. In one sample, chemical speciation was carried out because the concentration of As was below the detection limit [10]. We performed a conservative evaluation of the worst-case scenario by considering iAs to account for 100% of the total As (tAs). We also estimated the risk when considering iAs to account for only a fraction of the tAs, 52%, on the basis of the available data [6,9,10]. For this analysis, we selected only elements for which slope factor and reference dose had been developed by national and international health agencies (IARC, U.S. EPA, and OEHHA).

### 2.3. Selection of Essential and Non-Essential Elements for the Risk Assessment and Their Concentrations in Infant Cereal

Of the 22 chemicals identified, 5 have been assessed as carcinogens by the International Agency for Research on Cancer [33]: As, Cd, Al, and Ni are classified as group 1 agents (carcinogenic to humans); and Pb is classified as a group 2A agent (probably carcinogenic to humans). The IARC classifies Se and Cr as group 3 agents (not classifiable regarding their carcinogenicity to humans). The cancer slope factor is available for only two agents: iAs, for which the slope factor is 1.5 mg/kg per day [7]; and Pb, for which it is 8.5 × 10^−3^ mg/kg per day [34]. As shown in Table 2, reference doses are available for 15 of the elements identified.

Although there is evidence that Al, Cd, and Ni are carcinogenic, they were not included in our risk assessment because there are no slope factors available for those elements. Therefore, the ILCR for infant cereal intake is likely higher than that described in the present study.

## 3. Results

### 3.1. Exposure to Non-Essential Elements in Infant Cereal

Table 3 shows the concentrations of essential and non-essential elements in 18 different infant cereals, according to the main raw material of the infant cereal, as described by Pedron et al. [10]. Table 4 presents the maximum contaminant levels (MCLs) proposed by different agencies. When we considered iAs to account for 100% of the tAs in the eighteen samples evaluated, its concentration was above the Brazilian MCL in only one sample (5.5%), whereas it was above the MCLs proposed by the United States Food and Drug Administration (FDA) and the European Commission (EC) in eight (44.4%). However, for arsenic, most of the data to which we had access were related to the tAs in infant cereal, because the concentrations were so low that speciation proved impractical. Therefore, it is possible that the concentrations of iAs in all of the samples were below all three of the MCLs referenced. Pedron et al. [10] performed the speciation of iAs in one sample of rice cereal from Brazil (the one with the highest tAs concentration) and found that iAs accounted for approximately 40% of the tAs. Signes-Pastor et al. [9] found that the proportion of iAs in samples of rice cereal ranged from approximately 14% to approximately 90%. The FDA reported that, in 69 samples of infant cereal, the mean concentration of iAs was 120 ng g^−1^, corresponding to approximately 60% of the tAs [6]. If we considered iAs to account for 90% of the tAs, its concentration would be above the EC and FDA MCLs in seven samples (38.9%). If we considered iAs to account for 80%, 70%, 60%, and 50% of the tAs, its concentration would be above those MCLs in four (22.2%), two (11.1%), one (5.5%), and none of the eighteen samples, respectively.

According to the data obtained by Pedron et al. [10], the mean concentration of tAs was higher for rice cereals (111.37 ± 43.23 ng g^−1^; *n* = 9) than for multi-grain cereals containing rice (48.13 ± 46.76 ng g^−1^; *n* = 5) and non-rice-based cereals (7.86 ± 4.12 ng g^−1^; *n* = 4). That is in agreement with the findings of an independent study conducted in the United States, in which the mean concentration of iAs was 85 ng g^−1^ in rice cereal, compared with 23 ng g^−1^ in multi-grain cereal containing rice, 17 ng g^−1^ in multi-grain cereal with no rice, and 13 ng g^−1^ in oatmeal [49]. The FDA found the concentration of iAs to be 105 ng g^−1^ of in dry cereal made with white rice and 120 ng g^−1^ in dry cereal made with brown rice [6]. In Argentina, Londonio et al. [50] found the concentration of tAs to be 80.4 ng g^−1^ in one sample of rice cereal, although no statistical conclusions could be drawn.

The concentration of Cd was under the MCLs in all of the samples evaluated. However, two samples (one cereal containing rice and oats and one containing only rice) were found to have concentrations of Pb above the MCLs established for Brazil and for the European Union [2,51]. A more concise study conducted in Brazil evaluated the concentration of essential and toxic elements present in nine samples of infant cereals. The concentrations are generally aligned with the results reported by Pedron, except for Pb, which in this study exhibited concentrations 25 to 209 times higher. They also reported that the concentrations of the elements varied depending on the raw material [52]. In a study conducted in Spain [53], the concentrations of Pb in rice cereal for infants were found to be quite high (mean, 116 ± 37 ng g^−1^). The authors highlighted the need to intensify efforts to identify the sources of Pb and to reduce the levels of this contaminant in rice-based infant foods. Recently, the United Nations Food and Agricultural Organization ruled that the proposed provisional tolerable weekly intake (PTWI) for Pb could no longer be considered health-protective [54], which means that no level of Pb intake is considered safe.

**Table 3:** Concentrations of essential and non-essential elements in infant cereal [10].

Type of Infant Cereal (ID)	Essential Elements										Non-Essential Elements				
	Ag **	B **	Ba	Co	Cr	Cu	Mn	Se	Sr	Zn	As	Al **	Ni	Cd	Pb
	(ng g^−1^)	(ng g^−1^)	(ng g^−1^)	(ng g^−1^)	(ng g^−1^)	(ng g^−1^)	(ng g^−1^)	(ng g^−1^)	(ng g^−1^)	(ng g^−1^)	(ng g^−1^)	(ng g^−1^)	(ng g^−1^)	(ng g^−1^)	(ng g^−1^)
Corn (A) *	5 × 10^−5^ ± <0	5 × 10^−5^ ± <0	307.95 ± 29.6	6.45 ± 0.9	109.35 ± 13.1	1212.91 ± 252.1	2481.93 ± 222.1	62.01 ± 22.4	6191.67 ± 110.3	76,980.20 ± 3.07.2	4.75 ± 0.5	2258.88 ± 541.5	77.20 ± 13.6	0.77 ± 0.04	8.59 ± 1.7
Oatmeal (B) *	24.67 ± 16.9	135.03 ± 67.4	3228.01 ± 112.0	29.48 ± 6.9	90.08 ± 3.1	2663.27 ± 291.8	25,916.32 ± 847.3	6.83 ± 2.5	7350.02 ± 59.7	92,817.65 ± 2929.3	12.54 ± 0.6	1751.27 ± 125.0	498.52 ± 21.1	1.58 ± 2.0	26.66 ± 5.4
Oatmeal (C) *	5 × 10^−5^ ± <0	5 × 10^−5^ ± <0	2314.38 ± 79.9	11.69 ± 0.7	82.52 ± 8.1	3396.00 ± 321.8	27,957.60 ± 1485.2	53.31 ± 22.9	6856.69 ± 203.8	87,975.73 ± 3594.2	6.29 ± 1.3	3516.70 ± 322.6	417.23 ± 28.1	1.85 ± 0.7	16.63 ± 8.5
Multi-grain (D) *	5 × 10^−5^ ± <0	5 × 10^−5^ ± <0	1268.78 ± 71.6	11.62 ± 2.0	118.01 ± 22.8	1731.44 ± 222.5	8076.69 ± 296.5	37.05 ± 0.6	992.36 ± 38.0	132,895.81 ± 19,677.2	12.86 ± 2.5	2797.43 ± 612.9	82.26 ± 14.4	9.52 ± 3.2	31.30 ± 10.3
Rice and oat (E)	10.47 ±1.0	820.81 ± 122.2	580.32 ± 33.2	14.47 ± 3.3	339.80 ± 43.5	2939.32 ± 107.8	16,123.31 ± 4897.1	754.05 ± 166.5	7.99 ± <0	40,778.36 ± 753.1	26.32 ± 0.6	8799.73 ± 897.2	416.49 ± 18.5	3.43 ± 0.5	60.13 ± 2.3
Rice and oat (F)	11.79 ± 7.4	142.63 ± 104.2	1101.32 ± 24.3	19.86 ± 0.7	49.53 ± 0.4	1583.76 ± 103.6	10,347.66 ± 171.8	31.04 ± 2.8	47,843.81 ± 2192.5	66,817.50 ± 14,264.2	122.67 ± 5.5	3870.41 ± 852.0	182.15 ± 3.3	12.97 ± 1.1	25.52 ± 5.7
Rice and fruit (G)	32.16 ± 32.2	1908.55 ± 267.3	1211.96 ± 29.3	61.22 ± 2.1	209.50 ± 16.3	4225.63 ± 31.7	13,222.21 ± 489.4	45.06 ± 1.5	3246.27 ± 67.2	15,987.85 ± 682.6	90.98 ± 3.8	4723.68 ± 634.0	358.29 ± 16.3	14.27 ± 4.3	28.05 ± 6.8
Rice and cornstarch (H)	5 × 10^−5^ ± <0	5 × 10^−5^ ± <0	414.44 ± 84.7	14.46 ± 0.2	383.63 ± 77.7	3113.82 ± 118.1	13,189.60 ± 771.2	48.59 ± 22.3	16,535.69 ± 1147.9	67,200.60 ± 3539.6	18.92 ± 4.7	4670.31 ± 412.1	484.46 ± 21.3	4.33 ± 0.9	22.57 ± 5.4
Rice and cornstarch (I)	5 × 10^−5^ ± <0	5 × 10^−5^ ± <0	3.56 ± 0	5.30 ± 3.7	138.97 ± 12.4	202.41 ± 36.3	1022.39 ± 101.6	47.55 ± 43.6	85.15 ± 36.1	108,784.53 ± 10,816.7	17.05 ± 2.7	1264.50 ± 569.9	80.63 ± 20.2	3.06 ± 0.7	20.26 ± 14.9
Rice (J)	5 × 10^−5^ ± <0	5 × 10^−5^ ± <0	303.14 ± 9.8	11.93 ± 2.2	93.87 ± 7.9	1729.32 ± 493.7	7024.22 ± 138.6	26.90 ± 2.9	4649.73 ± 120.4	69,740.57 ± 1467.5	113.45 ± 6.0	5 × 10^−5^ ± <0	173.25 ± 6.4	2.07 ± 0.4	49.97 ± 15.5
Rice (K)	21.64 ± 2.0	5 × 10^−5^ ± <0	336.43 ± 45.1	23.34 ± 2.4	71.43 ± 8.0	2053.55 ± 51.9	10,304.56 ± 245.7	1001.25 ± 179.4	32.41 ± 13.2	121,475.24 ± 67,201.7	113.65 ± 10.6	552.00 ± 187.9	220.74 ± 13.5	17.14 ± 1.2	24.71 ± 8.8
Rice (L)	5 × 10^−5^ ± <0	5 × 10^−5^ ± <0	214.47 ± 16.1	27.82 ± 2.1	60.39 ± 2.5	2198.62 ± 165.6	11,485.59 ± 951.4	32.87 ± 11.0	775.21 ± 23.4	126,856.65 ± 57,523.7	125.70 ± 11.7	81.36 ± 10.0	172.05 ± 7.6	19.13 ± 3.5	32.04 ± 19.1
Rice (M)	32.90 ± 34.9	310.60 ± 176	567.04 ± 91.9	17.65 ± 0.7	266.53 ± 36.2	1888.35 ± 100.9	14,092.72 ± 384.1	28.10 ± 3.1	1128.08 ± 65.7	124,412.43 ± 30,568.1	146.27 ± 7.41	5 × 10^−5^ ± <0	183.32 ± 1.9	22.15 ± 4.0	20.52 ± 9.1
Rice (N)	27.39 ± 9.9	1062.21 ± 405.2	148.01 ± 8.4	24.54 ± 1.6	69.97 ± 31.5	1953.83 ± 215.5	9080.14 ± 318.7	47.81 ± 10.0	123.22 ± 4.2	114,733.40 ± 5848,0	102.36 ± 5.9	2409.44 ± 978.2	112.79 ± 12.4	13.87 ± 7.2	22.67 ± 18.4
Rice (O)	64.63 ± <0	0.00 ± <0	422.87 ± 36.1	74.73 ± 5.2	180.24 ± 101.2	2928.29 ± 649.3	15,125.22 ± 943.9	53.46 ± 32.0	177.21 ± 26.4	106,956.31 ± 16,955.2	168.08 ± 29.0	865.44 ± 243.2	264.91 ± 48.3	18.71 ± 11.3	15.58 ± 5.6
Rice (P)	17.01 ± 7.3	1572.62 ± 233.1	1192.17 ± 20.9	54.56 ± 5.0	211.67 ± 18.5	4299.29 ± 309.4	14,414.66 ± 955.9	42.69 ± 7.26	3959.14 ± 211.4	57,728.65 ± 11,198.9	86.71 ± 6.1	4961.73 ± 413.3	432.94 ± 50.1	10.86 ± 1.3	16.37 ± 13.3
Rice (Q)	48.57 ± 0	5 × 10^−5^ ± < 0	441.57 ± 32.4	33.17 ± 5.4	38.36 ± 2.8	2866.51 ± 367.8	9949.67 ± 679.4	13.54 ± 1.51	6923.74 ± 296.0	53,445.06 ± 3691.6	130.85 ± 6.9	3549.55 ± 500.4	214.89 ± 12.2	12.52 ± 9.8	33.19 ± 18.8
Rice (R)	1.09 ± 0.24	5 × 10^−5^ ± <0	56.21 ± 11.9	14.80 ± 4.1	211.16 ± 23.3	335.09 ± 145.0	1616.48 ± 74.8	826.97 ± 232.2	5.21 ± 5.6	13,0184.69 ± 68,389.0	15.27 ± 0.7	5 × 10^−5^ ± <0	78.03 ± 11.2	2.29 ± 1.3	31.24 ± 9.9

Ag = silver; Al = aluminum; As = arsenic; ATSDR = Agency for Toxic Substances and Disease Registry; B = boron; Ba = barium; Cd = cadmium; Co = cobalt; Cr = chromium; Cu = copper; FAO = (United Nations) Food and Agricultural Organization; IRIS = (U.S. Environmental Protection Agency) Information Risk Information System; Mn = manganese; Ni = nickel; OEHHA = (California) Office of Environmental Health Hazard Assessment; Pb = lead; RfD = reference dose; Se = selenium; Sr = strontium; Zn = zinc.* Non-rice-based cereal.** Limit of detection = 5 × 10^−5^ ng g^−1^.

**Table 4:** Maximum contaminant levels of metals in infant cereal, according to different agencies.

Country	Maximum Contaminant Level *			Reference(s)
	iAs	Total Cd	Total Pb	
	(ng g^−1^)	(ng g^−1^)	(ng g^−1^)	
Brazil	150	50	50	Ministry of Health [51]
United States	100	-	-	U.S. FDA [6]
European Union	100 *	40	50	EC [55,56,57]

Cd = cadmium; iAs = inorganic arsenic; Pb = lead. * For rice destined for the preparation of foods for infants and children.

### 3.2. Cancer Risk of Exposure to Non-Essential Elements in Infant Cereal

**Figure 1:**
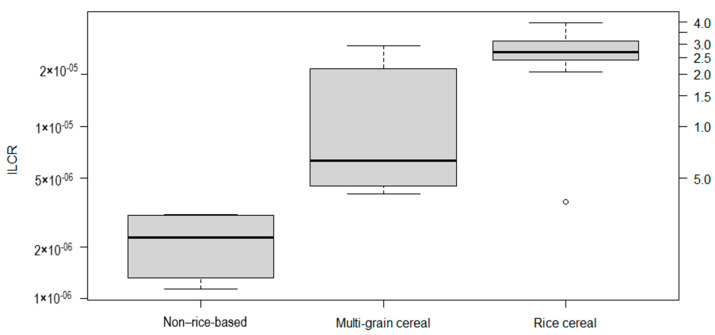
Box plot of the incremental lifetime cancer risk (ILCR) associated with exposure to inorganic arsenic and lead in infant cereal, by composition, on a logarithmic scale.

Table S1 (in the Supplemental Material) presents the incremental cancer risks by age group, together with the ILCR for iAs, Pb, and the combination of the two. Figure 1 presents the box plot of the total ILCR (the outlier represented as a dot). On the basis of the data related to the samples evaluated, we found that the ILCR for exposure to iAs and Pb combined ranged from 1.14 × 10^−6^ (for corn cereal) to 3.99 × 10^−5^ (for rice cereal). The estimated cancer risk was higher for the 12 to <24 months age group, in which the average daily dose was approximately double that estimated for the 4 to <12 months age group (Table S2). For infants 12 to <24 months of age, the average daily ingestion of rice was estimated to be 8 g/kg of BW, compared with 4 g/kg of BW for those 4 < 12 months of age [30,32]. Minimizing the exposure by delaying weaning until the infant reaches 12 months of age could reduce the cancer risk by 24%.

There are no guidelines for the acceptable risk related to carcinogenic contaminants in food. However, if we considered the acceptable risk level suggested by the WHO [58] for carcinogenic compounds in drinking water (10^−5^), as did authors such as Shibata et al. [4], the mean ILCR for rice cereal in our study would be 2.6 times higher than that level, and multi-grain cereal containing rice would be 1.3 times higher. The non-rice-based cereal would be the only category whose estimated ILCR value is below 10^−5^, being 2.2 × 10^−6^.

Regarding the type of infant cereal, we found that the ILCR was highest for rice cereals (mean, 2.6 × 10^−5^ ± 9.7 × 10^−6^), followed by multi-grain cereals containing rice (mean, 1.3 × 10^−5^ ± 1.0 × 10^−5^) and non-rice-based cereals (mean, 2.2 × 10^−6^ ± 8.7 × 10^−7^), as presented in Figure 1. The higher risk associated with rice cereal intake can be attributed to the high concentrations of iAs that are typical of rice and rice products. Rice is known to store higher levels of As than do other crops [59]. Toledo et al. [60] found a high cancer risk associated with rice consumption in Brazil due to iAs exposure. In the present study, the mean ILCR for rice cereal was two times higher than that for multi-grain cereal, and approximately 12 times higher than that for non-rice-based cereal. Signes-Pastor et al. [9] found a good correlation between the iAs concentration and rice content in infant cereal, demonstrating that most of the iAs comes from rice. We found some variability in the risk results, even among the samples of rice cereal, which can be related to the proportion of rice in relation to other cereals, as well as to the origin of the rice and the manufacturing process [9]. In our study, iAs contributed the largest portion of the ILCR in all samples, including the non-rice-based cereal samples, ranging from 98.5% to 99.9% of the total ILCR. This is not surprising, as iAs has a high cancer slope factor (1.5 mg/kg day^−1^), which is more than 176 times higher than that of Pb (8.5 × 10^−3^ mg/kg day^−1^).

Infant cereal can represent the main source of iAs for infants and children, as suggested by Shibata et al. [4], in a study conducted in the USA. Those authors found that the ILCR from dietary exposure to iAs during infancy was 10^−5^ and that rice cereal accounted for 55% of the dose of iAs in infants between 4 and 24 months of age, compared with 19% for other solid infant foods, 18% for drinking water, and 9% for infant formula. The concentration of As in foodstuff is influenced by the food type, growing conditions, and food-processing techniques.

In the present risk assessment, we did not include organic arsenic in the risk calculations, since there is little available information about its cancer and non-cancer effects. The IARC classifies organic arsenic as a group 3 agent (not classifiable regarding its carcinogenicity). In its risk assessment of exposure to As in rice and rice products, the FDA collected evidence about organic As from the literature, which indicated that exposure to some organic As compounds could be related to effects on the bladder, kidneys, thyroid, and gastrointestinal tract, as well as on fetal development. However, a reference dose for this compound has not yet been developed, which has led to the exclusion of organic arsenic from the current study [6]. Thus, the actual risk could be higher than we estimated. In addition, we had access only to data about the tAs rather than iAs, which would be more appropriate because the available slope factor was developed for iAs. The reported proportion of iAs in infant cereals ranges from 14% to 90%, and the mid-point of this proportion is 52% [6,10,61]. In order to perform a risk assessment with less uncertainty, we estimated the cancer risk assuming that iAs accounted for 52% of the tAs, as described in Table S3. Under that assumption, the estimated risk related to iAs in infant cereal was found to range from 5.9 × 10^−7^ to 2.1 × 10^−5^, which is, as expected, 52% of the initial estimate. That indicates how speciation of iAs is important.

Because our risk assessment considered exposure only for one life stage and one type of food, the actual risk might be higher. Drinking water and other types of food usually consumed in certain regions have been reported to be major sources of exposure to As, including fish, seafood, and algae-based food products or supplements [62,63].

In the present study, the incremental lifetime cancer risk for exposure to Pb in infant cereal ranged from 1.15 × 10^−8^ (for corn cereal) to 8.08 × 10^−8^ (for cereal containing rice and oats). The mean ILCR was higher for multi-grain cereal than for rice cereal and non-rice-based cereal (4.2 × 10^−8^ vs. 3.7 × 10^−8^ and 2.8 × 10^−8^, respectively). The ILCR for exposure to Pb is considered low in comparison with the WHO standard for drinking water [58] and is significantly lower than that related to iAs.

Neto et al. [64] conducted a systematic review and meta-analysis with the main goal of estimating the Pb content in food produced or consumed in Brazil. They found that infant food, which includes infant cereal, was the food category in which the mean concentration of Pb is the highest (0.48 mg/kg). The authors also identified relevant uncertainty about the results related to infant food, mainly because of a lack of data. Leroux et al. [65] found that the diets of preschool children in Brazil did not contain levels of As and Pb higher than those of children in other countries. However, given the overall exposure, the authors stated that diet may contribute significantly to health risks.

### 3.3. Non-Cancer Risk of Exposure to Essential and Non-Essential Elements in Infant Cereal

**Figure 2:**
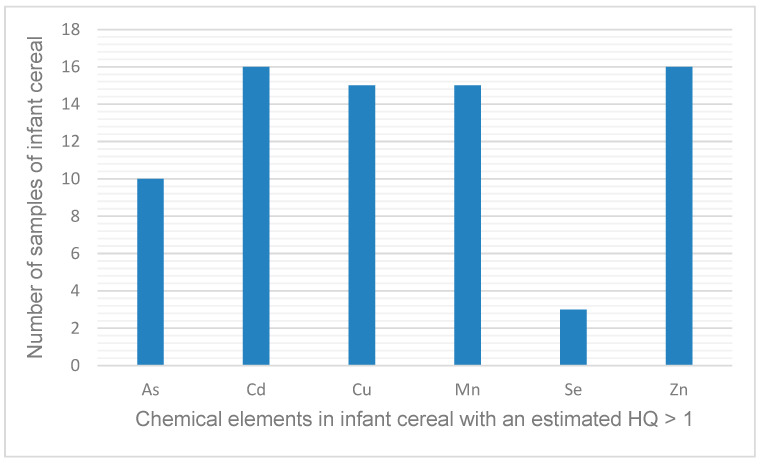
Elements present in infant cereal and the number of samples in which the estimated hazard quotient (HQ) was >1 for a given element.

Table S4 shows the results of the non-cancer risk assessment for each element and cereal evaluated. The elements with an HQ > 1 are presented in Figure 2.

All of the infant cereals evaluated had an HQ > 1 for at least one element (Table S4), which means that the daily dose is higher than the reference dose that estimates daily oral exposure to the human population (including sensitive subpopulations) that is likely to be without an appreciable risk of deleterious effects during a lifetime. As shown in Figure 2, six elements (As, Cd, Cu, Mn, Se, and Zn) were responsible for the HQs > 1. The elements that were responsible for an HQ > 1 in the most samples (*n* = 15 or 16) were Cd, Cu, Mn, and Zn, whereas Se was responsible for an HQ > 1 in the fewest samples (*n* = 3).

In the present study, the proportion of elements with an HQ > 1 was 81.5% for rice cereal, compared with 52.8% for multi-grain cereal and 50.0% for non-rice-based cereal. A study evaluating more samples of infant cereal could provide a more sophisticated statistical analysis.

Some of the chemicals with an HQ > 1 are essential elements that are needed by the human body only in small amounts (Cu, Mn, Se, and Zn), and toxic effects may occur if the dose exceeds certain levels [66]. In general, the excessive presence of essential elements in infant cereals is predominantly attributed to artificial fortification by the industry aimed at enhancing the nutritional profile of infant cereals [25]. In a review of the literature, Garcia-Casal et al. [25] reported that excessive intake of certain essential elements through the ingestion of fortified food might represent a health risk, especially in countries where there are simultaneous micronutrient-delivery interventions. The authors also stated that it is a challenge to establish recommendations regarding the maximum amount of certain essential elements to be allowed in food products, and risk management could be an important tool to support the decision-making process. Another aspect to consider is that the levels of certain nutrients can be significantly higher in infant cereal than in breast milk, and that can be a cause for concern because of the high proportion of infants that are introduced to infant cereal before 6 months of age [67].

Ljung et al. [68] evaluated different kinds of infant food and found that the intake of As by infants 4 months of age varied greatly among the different foods, and was 1–95 times higher for such foods than for breast milk. Carbonell-Barrachina et al. [53] called attention to the diets of individuals with celiac disease, which is richer in iAs than that of the general population. The authors found that gluten-free infant food usually contains rice and therefore a higher concentration of iAs. The non-cancer effects related to oral exposure to iAs are skin lesions and diabetes, together with effects on the cardiovascular and immune systems [69]. In our study, the lack of data regarding iAs in the samples is a limitation, because the reference dose is attributed to iAs, rather than to the tAs. If we consider iAs to account for 52% of the tAs, following the same assumptions made for the cancer risk estimation in Section 3.2, the HQs would be lower, as described in Table S5. Even with the HQ values representing only 52% of the previous estimations, the number of samples with an HQ > 1 remains the same. Therefore, 10 samples of infant cereal (cereal containing rice and oats, rice cereal with fruit, and cereal containing only rice) might present risks to human health.

Toxic minerals or trace elements may be present in infant food primarily because of their natural occurrence in the raw materials [36]. The non-essential element with a relevant risk in the greatest number of cereal samples was Cd. Dietary exposure to Cd can result in kidney injury and bone demineralization. It can be retained in the kidney and liver with a biological half-life of 10–30 years [19]. In a study assessing various types of infant food, Carbonell-Barrachina et al. [53] found that pure baby rice, which is normal rice ground to a powder, had the highest Cd concentration. The levels of Cd can be higher in cereal-based food than in milk-based food [67]. Ljung et al. [68] estimated the intake of Cd by infants 4 months of age to be 3–270 times higher for infant food than for breast milk. In France, Jean et al. [70] found that a high proportion of children exceed the tolerable weekly Cd intake of 2.5 μg/kg BW^−1^ proposed by the European Food Safety Authority, and that infant cereals were among the main contributors to that. Olympio et al. [71] found blood Cd levels to be almost twelve times higher among preschool children in Brazil than among those in the United States, a country with strong public policies related to chemical exposure. In their cross-sectional study, those authors included 2463 children at 50 day care centers in the city of São Paulo, and there was an association between blood cadmium levels and day care center geographic regions, indicating hot spots for cadmium exposure. In the present study, the non-cancer risk was found to be higher for rice cereal, for which an average of 4.7 elements resulted in an HQ > 1, compared with 3.5 elements for non-rice-based cereal.

Avoiding the intake of infant cereal among infants 4–12 months of age could reduce the number of elements with an HQ > 1. That would apply to Cu (5 samples) and Se (2 samples). According to the WHO, replacing breastfeeding with inappropriate supplementary feeding, especially in the first six months of life, can be characterized as a risk factor for morbidity and mortality, the long-term effects being poor school performance and lower productivity, as well as limited intellectual and social development [72]. Breastfeeding can reduce the risk of type 2 diabetes and overweight/obesity [73]. Exclusive breastfeeding in early life has a protective effect against gastrointestinal and allergic diseases [74].

Among the samples evaluated in the present study, the HQ was < 1 for Ag, B, Ba, Co, Cr, Ni, Sr, Al, and Pb. The HQ for Pb was calculated on the basis of the reference dose estimated from the previous PTWI [54]. The PTWI was withdrawn because it was concluded that it could no longer be considered health-protective [75]. Therefore, there is no level of Pb exposure that is currently considered safe. Carbonell-Barrachina et al. [53] reported that, among various types of infant food, rice cereals had the highest concentrations of Pb. Added ingredients, such as cocoa, honey, and fruit, can also be sources of some metals, such as Cd and Pb, in infant cereal [12].

## 4. Discussion

Important measures to mitigate risk are mainly aimed at reducing the concentrations of chemicals in infant cereals or at reducing the intake of such cereals. In their risk assessment, the FDA proposed three hypothetical scenarios in which mitigation measures could be taken [6]: establishing limits for iAs in infant cereals containing rice; changing the frequency of consumption of such cereals; and a combination of the two measures, which would result in the most significant reduction in iAs intake (from 153 ng/kg BW/day to ≤32 ng/kg BW/day). In this context, the FDA has an initiative called Closer to Zero that aims to reduce consumers’ exposure to toxic heavy metals such as Pb, Hg, Cd, and As in baby and toddler food. The initiative includes a series of actions, such as implementing stricter limits for the presence of these metals in food, encouraging the food industry to develop production methods that minimize contamination, providing information to consumers, and cooperating with international partners to ensure that imported foods also meet these standards [76]. The FDA stated that it is possible to reduce exposure to iAs from rice cereal through the use of current good manufacturing practices, highlighting the selection of rice with a low iAs content, and proposing an action level of 100 µg/kg (100 ng g^−1^) in infant rice cereals [77]. Carey et al. [78] reported that, to meet the standard of 100 ng g^−1^ of iAs in infant food proposed by the European Commission in 2016 [79], manufacturers were diluting rice with other gluten-free cereals. Hernández-Martínez and Navarro-Blasco [12] reported that some cereal producers in Spain have achieved considerable reductions in the concentrations of non-essential elements, likely by being more selective in their choice of raw materials and preventing contamination during industrial manufacturing. Among the rice cereal samples evaluated in the present study, the mean concentration of tAs was 111.4 ± 43.2 ng g^−1^ (95% confidence interval, 78.14–144.6), only slightly higher than the 100 ng g^−1^ proposed by the FDA for iAs. It is possible that As speciation would reveal a concentration of iAs below the proposed standard. There is significant variability in As concentration, and the small number of samples is not representative of the rice-based infant cereals consumed in Brazil. Nonetheless, if we consider the scenario where iAs concentration in the samples included in this study correspond to 52% of total As, the mean ILCR would be 1.4 × 10^−5^, above the drinking water action level (10^−5^). Therefore, our results suggest that even a maximum contaminant level of 100 ng g^−1^, despite bringing important benefits to public health, would still have some residual risk in the Brazilian context. In addition, with the advancement of discussions about the adverse health effects resulting from exposure to organic arsenic, this should also be considered in future studies.

The intake of rice cereal could be reduced by varying the type of infant cereal offered to infants and children. As shown in Table 4 and Table S1, different types of cereal contain different concentrations of a variety of elements and can present distinct levels of risk. The type of cereals given to infants varies according to the culture of a country. Rice, oat, wheat, and cornstarch cereals appear to be the most popular [1]. Infants and children usually have a less varied diet than do adults, which increases the level of concern about exposure to non-essential elements and health risks. Infants and children are also a more vulnerable population because they consume more food in proportion to their body weight than do adults [77].

There have been a few previous studies investigating the kind of infant cereal consumed by infants and children in Brazil. In a study of 63 infants 0–6 months of age in a peripheral area in northeastern Brazil, Albuquerque et al. [80] reported that the infants usually consumed more than one kind of infant cereal, rice cereal being consumed by >76%, whereas 29% consumed cornstarch cereal, 10% consumed multi-grain cereal (containing wheat, barley, and oats), and 3% consumed oatmeal. Approximately 90% of the infants consumed 2–8 portions of infant cereal, infant formula, or whole milk per day. In a cross-sectional study of 60 children (4 to 36 months of age) born into low- or middle-income families in the northeastern Brazilian state of Ceará, Sombra et al. [81] found rice cereal to be the most common choice of infant cereal among children 4–36 months of age (*n* = 60), accounting for 27% of the children, followed by corn cereal (20%), wheat cereal (10%), cornstarch (7%), oatmeal (5%), and multi-grain cereal (3%).

Other measures that can be taken to mitigate the risk of exposure to non-essential elements in rice cereals include adopting good agricultural practices and monitoring soil/water contamination. The concentration of iAs in rice can vary significantly depending on agricultural practices and environmental conditions. In Brazil, Kato et al. [82] found a difference of two orders of magnitude in rice samples grown under different conditions related to water, at different locations, and from different cultivars. In southern Brazil, the main rice-producing region of the country, Monteiro et al. [83] found significant differences in the accumulation of essential and non-essential elements in rice. They attributed it primarily to environmental conditions and secondarily to the rice variety. Segura et al. [84] emphasized the importance of crop tracking to identify rice with a low iAs concentration and use it to produce food for vulnerable populations, such as infants, children, and individuals with celiac disease. In an exploratory study conducted in Brazil, Lange et al. [85] successfully traced rice sources by city, producer, and rice variety.

Regarding the excess of essential elements in infant rice, Garcia-Casal et al. [25] stated that there is a significant lack of data about intakes and actual deficiencies in the population. Although they acknowledged that adding essential elements to infant food can be an important measure for public health, they suggested that, in some situations, infants are receiving more than is necessary. The authors recognized the challenge posed by this situation and highlighted the need for surveillance, monitoring, and evaluation systems that are more accurate.

Reducing the concentrations of elements in infant cereal or increasing the variety of infant cereals in the diet of infants probably would not solve the problem if the infants were weaned early. In the present study, we assumed that infants would start consuming infant cereal at 4 months of age, because many studies have reported early interruption of breastfeeding and have shown that infant formula and infant cereal are the foods typically chosen to replace breast milk in Brazil [80,81].

In the study conducted by Albuquerque et al. [62,80], in a peripheral area in northeastern Brazil, the authors found that exclusive breastfeeding was discontinued before 1 month of age in 43% of the infants and by 4 months of age in the remainder. Among the 63 infants evaluated, infant cereal was the replacement for breast milk in 58.7% and the most common period during which such cereal was consumed was from 0 to 4 months of age.

In their cross-sectional study in the northeastern Brazilian state of Ceará, Sombra et al. [81] found that 10% of the children were introduced to some type of complementary food before 4 months of age. The main reason was the need for mothers to return to work. The authors identified a relevant prevalence of infant cereal among the complementary foods.

The WHO recognizes that following the guidance that exclusive breastfeeding should be maintained until an infant reaches 6 months of age and that breastfeeding should be continued, together with complementary foods, until the infant is at least 24 months of age can be a challenge [86]. This is a global issue, given that exclusive breastfeeding is maintained throughout the first 6 months of life in fewer than 40% of infants worldwide [87]. In Brazil, a maternity leave of 120 days is guaranteed for women who are in the formal labor market [88]. For those engaged in informal labor and other formal work arrangements, the number of workers who do not have labor rights, including maternity leave, is on the rise [89]. Monteiro et al. [90] conducted a cross-sectional study with data from a national survey and found evidence that the lack of maternity leave in Brazil results in a 23% increase in the chance that exclusive breastfeeding will be discontinued for infants under 4 months of age. In another cross-sectional study, conducted in the city of Rio de Janeiro, Brazil, Rimes et al. [91] found the prevalence of exclusive breastfeeding in infants up to 6 months of age to be 50.1% and to be associated with access to maternity leave.

Among mothers in Latin America, some reported reasons for early discontinuation of breastfeeding are lack of support from family, previous experiences, psychological aspects, the need to return to work, and breast problems related to breastfeeding, as well as a lack of time. Some mothers also have the mistaken idea that breast milk is not sufficient to feed their infants [92,93,94].

Some governmental initiatives promote maintaining exclusive breastfeeding for up to 6 months through educational programs and the creation of organized groups of mothers to exchange experiences for mutual support. There is evidence that these initiatives have been effective [95,96]. Another strategy adopted by the Brazilian government is providing incentives for public and private companies to offer day care at the workplace and to have a breastfeeding support room [97,98,99], and well as giving lactating mothers the right to two extra breaks to breastfeed [100]. A national law created in 2008 guarantees that civil servants have 180 days of maternity leave and 20 days of paternity leave, the latter being only 5 days for employees of privately held companies. The same conditions are guaranteed for employees of companies that adhere to the program stimulated by tax incentives [101]. However, adherence by the private sector is low; in 2016, only approximately 10% of the companies in Brazil committed to the program [101]. Therefore, it is common that after the maternity leave ends, the infants start to spend the day at day care centers, at which point breastfeeding is typically discontinued. In the city of São Paulo, the food consumption guidelines for day care centers of the municipality recommend that infants 0–3 months of age receive infant formula and those ≥4 months of age be introduced to vegetables, rice, meat, and fruit [102].

Delaying infant cereal consumption in the first months of life might reduce the exposure to infants to certain elements present in this kind of food, and possibly the health risks. In addition to maintaining breastfeeding until the infant reaches 6 months of age, which is not always a choice, complimentary foods could be introduced as part of a balanced diet with the support of public policies.

At the present moment in Brazil, no publication addressing the risks outlined in this study has been observed, nor has there been any regulatory effort to encourage a reduction in the consumption of infant cereals. This gap in both research and regulatory initiatives underscores the need for further exploration into the potential health implications associated with infant cereal consumption and highlights the importance of implementing regulatory measures to promote safer feeding practices for infants. Addressing this gap is crucial to ensuring the health and well-being of infants and underscores the necessity of comprehensive regulatory frameworks that prioritize infant nutrition and safety.

## 5. Conclusions

According to our risk assessment, rice cereal was the infant cereal that presents the highest cancer risk in Brazil, also having the most elements with an HQ > 1. The higher cancer risk is attributed to the typically high concentrations of iAs in rice.

We found that iAs was the element associated with the highest cancer risk, even for non-rice-based cereals. For the non-cancer risk, Zn and Cd were the most significant essential and non-essential elements, respectively. Although the cancer and non-cancer risks for Pb are relatively low, exposure to this element is always concerning, especially in a vulnerable population. Delaying the introduction of infant cereals until one year of age can reduce such risks. We emphasize the importance of breastfeeding, which, in addition to its known benefits, can be a safer food source in terms of avoiding non-essential elements and excess intake of essential elements. The literature suggests a complex scenario regarding delaying weaning and avoiding early exposure, involving maternity leave, culture, and agricultural and industry practices. Some studies and governmental initiatives have provided guidance in addressing the risk, and public polices in Europe and the U.S.A have succeeded in reducing the concentrations of iAs in infant cereal. An important finding of this study is that all samples contained at least one element with an HQ > 1, indicating a potentially more complex risk scenario in terms of risk management, with significant implications for the actions to be adopted.

Further studies are warranted to elucidate which regulatory measures and public policies are most suitable for addressing this issue, given its complex nature.

Our analyses would be strengthened with more data on different types of cereal collected from different parts of the country, as this would enable a probabilistic risk assessment, which is a more sophisticated and robust approach. The actual risk for infants is probably higher than we reported since we excluded from our assessment elements for which cancer slope factors and reference doses were unavailable, and we did not consider combined exposures. Actual risk would also depend on other types of contaminants and background cancer risks. Our results could have been different if we had included other kinds of food consumed by infants (such as infant formula) and drinking water, which are potential sources of iAs.

## Figures and Tables

**Table 1:** Parameters for the risk assessment.

Parameter	Age Group		Reference(s)
	4 to <12 Months	12 to <24 Months	
Body weight (kg)	7.8	11.2	IBGE [30]
Exposure frequency (days/year)	365	365	Shibata et al. [4]
Exposure duration (years)	0.67	1	Shibata et al. [4]
Ingestion rate (g/day)	31.2	94.08	IBGE; U.S. EPA [31,32]

**Table 2:** Toxicity values of the elements included in the risk assessment.

Element	RfD	Data Source
	(mg/kg·day)	
Ag	5.0 × 10^−3^	IRIS [35]
Al	1 *	ATSDR [36]
As	3.0 × 10^−4^	IRIS [7]
B	2.0 × 10^−1^	IRIS [37]
Ba	2.0 × 10^−1^	IRIS [38]
Cd **	1.1 × 10^−5^	OEHHA [39]
Co	0.01 *	ATSDR [40]
Cr	1.50	IRIS [41]
Cu	0.01 *	ATSDR [42]
Mn **	3.0 × 10^−2^	OEHHA [43]
Ni **	1.1 × 10^−2^	OEHHA [44]
Pb ***	3.5 × 10^−3^	FAO [45]
Se	5.0 × 10^−3^	IRIS [46]
Sr	6.0 × 10^−1^	IRIS [47]
Zn	3.0 × 10^−1^	IRIS [48]

RfD = reference dose; MRL = minimal risk level; Ag = silver; Al = aluminum; As = arsenic; ATSDR = Agency for Toxic Substances and Disease Registry; B = boron; Ba = barium; Cd = cadmium; Co = cobalt; Cr = chromium; Cu = copper; FAO = (United Nations) Food and Agricultural Organization; IRIS = (U.S. Environmental Protection Agency) Information Risk Information System; Mn = manganese; Ni = nickel; OEHHA = (California) Office of Environmental Health Hazard Assessment; Pb = lead; Se = selenium; Sr = strontium; Zn = zinc. * Minimal Risk Level (MRL). ** Child-specific RfD. *** RfD calculated according to the FAO provisional tolerable weekly intake, which has been withdrawn [45].

## Data Availability

The original contributions presented in the study are included in the article/Supplementary Materials. Further inquiries can be directed to the corresponding author.

## Supplementary Materials

The following supporting information can be downloaded at: https://www.mdpi.com/article/10.3390/ijerph21040381/s1, Table S1: Estimated incremental cancer risks and incremental lifetime cancer risks associated with exposure to non-essential elements present in different kinds of infant cereal; Table S2: Estimated average daily dose associated with exposure to essential and non-essential elements present in different kinds of infant cereal; Table S3: Estimated incremental lifetime cancer risk associated with exposure to non-essential elements present in different kinds of infant cereal, if inorganic arsenic is assumed to account for 52% of the total arsenic; Table S4: Fractional and lifetime hazard quotients for exposure to essential and non-essential elements present in infant cereal; Table S5: Estimated hazard quotients for exposure to inorganic arsenic, if it is assumed to account for 52% or 100% of the total arsenic.

## References

[1] Klerks M., Bernal M.J., Roman S., Bodenstab S., Gil A., Sanchez-Siles L.M. (2019). Infant Cereals: Current Status, Challenges, and Future Opportunities for Whole Grains. Nutrients.

[2] EC Commission Regulation (EU) (2006). No. 1881/2006 of 19 December 2006 Setting Maximum Levels for Certain Contaminants in Foodstuffs. Off. J. Eur. Union.

[3] Paiva E.L., Morgano M.A., Arisseto-Bragotto A.P. (2019). Occurrence and Determination of Inorganic Contaminants in Baby Food and Infant Formula. Curr. Opin. Food Sci..

[4] Shibata T., Meng C., Umoren J., West H. (2016). Risk Assessment of Arsenic in Rice Cereal and Other Dietary Sources for Infants and Toddlers in the U.S. Int. J. Environ. Res. Public Health.

[5] U.S. EPA (2019). Updated Problem Formulation and Protocol for the Inorganic Arsenic IRIS Assessment [CASRN 7440-38-2].

[6] U.S. FDA (2016). Arsenic in Rice and Rice Products Risk Assessment Report.

[7] U.S. EPA (1995). Chemical Assessment Summary: Arsenic (Inorganic).

[8] Zhao F.-J., McGrath S.P., Meharg A.A. (2010). Arsenic as a Food Chain Contaminant: Mechanisms of Plant Uptake and Metabolism and Mitigation Strategies. Annu. Rev. Plant Biol..

[9] Signes-Pastor A.J., Carey M., Meharg A.A. (2016). Inorganic Arsenic in Rice-Based Products for Infants and Young Children. Food Chem..

[10] Pedron T., Segura F.R., da Silva F.F., de Souza A.L., Maltez H.F., Batista B.L. (2016). Essential and Non-Essential Elements in Brazilian Infant Food and Other Rice-Based Products Frequently Consumed by Children and Celiac Population. J. Food Compos. Anal..

[11] Hernandez F., Bemrah N., Séby F., Noël L., Guérin T. (2019). Cr(VI) and Cr(III) in Milk, Dairy and Cereal Products and Dietary Exposure Assessment. Food Addit. Contam. Part B Surveill..

[12] Hernández-Martínez R., Navarro-Blasco I. (2012). Estimation of Dietary Intake and Content of Lead and Cadmium in Infant Cereals Marketed in Spain. Food Control.

[13] NRC (2013). Critical Aspects of EPA’s IRIS Assessment of Inorganic Arsenic: Interim Report.

[14] IARC (2012). Arsenic and Arsenic Compounds Monograph.

[15] ATSDR (2007). Toxicological Profile for Lead.

[16] Tchounwou P.B., Yedjou C.G., Patlolla A.K., Sutton D. (2012). Heavy Metal Toxicity and the Environment. Molecular, Clinical and Environmental Toxicology.

[17] WHO (2012). WHO List of Classifications.

[18] EFSA (2009). Scientific Opinion of the Panel on Contaminants in the Food Chain on a Request from the European Commission on Cadmium in Food.

[19] WHO 10 Chemicals of Public Health Concern. https://www.who.int/news-room/photo-story/photo-story-detail/10-chemicals-of-public-health-concern.

[20] Jan A.T., Azam M., Siddiqui K., Ali A., Choi I., Haq Q.M.R. (2015). Heavy Metals and Human Health: Mechanistic Insight into Toxicity and Counter Defense System of Antioxidants. Int. J. Mol. Sci..

[21] Zhang N., Baker H.W., Tufts M., Raymond R.E., Salihu H., Elliott M.R. (2013). Early Childhood Lead Exposure and Academic Achievement: Evidence from Detroit Public Schools, 2008–2010. Am. J. Public Health.

[22] (2010). WHO Childhood Lead Poisoning. World Health Organ..

[23] Olympio K.P.K., Oliveira P.V., Naozuka J., Cardoso M.R.A., Marques A.F., Günther W.M.R., Bechara E.J.H. (2010). Surface Dental Enamel Lead Levels and Antisocial Behavior in Brazilian Adolescents. Neurotoxicol. Teratol..

[24] Olympio K.P.K., Gonçalves C., Günther W.M.R., Bechara E.J.H. (2009). Neurotoxicity and Aggressiveness Triggered by Low-Level Lead in Children: A Review. Rev. Panam. Salud Publica/Pan Am. J. Public Health.

[25] Garcia-Casal M.N., Mowson R., Rogers L., Grajeda R. (2019). Risk of Excessive Intake of Vitamins and Minerals Delivered through Public Health Interventions: Objectives, Results, Conclusions of the Meeting, and the Way Forward. Ann. N. Y. Acad. Sci..

[26] EC Commission (2006). Directive 2006/125/EC on Processed Cereal-Based Foods and Baby Foods for Infants and Young Children. https://eur-lex.europa.eu/legal-content/EN/ALL/?uri=CELEX%3A32006L0125.

[27] FAO (2015). Guidance for Risk Management Options in Light of Different Risk Assessment Outcomes.

[28] U.S. EPA (1989). Risk Assessment Guidance for Superfund Volume I Human Health Evaluation Manual (Part A).

[29] U.S. EPA (2005). Supplemental Guidance for Assessing Susceptibility from Early-Life Exposure to Carcinogens.

[30] de Crianças A.e.E.N., Brasil A.e.A.N. (2010). IBGE Pesquisa de Orçamentos Familiares 2008–2009.

[31] IBGE (2020). Pesquisa de Orçamentos Familiares 2017–2018: Análise de Consumo Alimentar Pessoal No Brasil.

[32] U.S. EPA (2009). Child-Specific Exposure Factors Handbook.

[33] IARC (2020). Agents Classified by the IARC Monographs.

[34] OEHHA (2020). Appendix A: Hot Spots Unit Risk and Cancer Potency Values.

[35] U.S. EPA (1991). Reference Dose for Chronic Oral Exposure of Silver.

[36] ATSDR (2008). Toxicological Profile for Aluminium.

[37] U.S.EPA (2004). Chemical Assessment Summary: Boron and Compounds.

[38] US. EPA (2005). Chemical Assessment Summary: Barium and Compounds.

[39] OEHHA—Office of Environmental Health Hazard Assessment (2005). Cadmium.

[40] ATSDR (2004). Toxicological Profile for Cobalt.

[41] US. EPA (1998). Chemical Assessment Summary: Chromium (III), Insoluble Salts.

[42] ATSDR (2004). Toxicological Profile for Copper.

[43] OEHHA (2006). Manganese and Pentachlorophenol.

[44] OEHHA—Office of Environmental Health Hazard Assessment (2005). Nickel and Nickel Compounds.

[45] Joint FAO/WHO (2011). Evaluation of Certain Food Additives and Contaminants.

[46] U.S. EPA (1991). Chemical Assessment Summary: Selenium and Compounds.

[47] U.S. EPA (1992). Chemical Assessment Summary: Strontium.

[48] U.S. EPA (2005). Chemical Assessment Summary: Zinc and Compounds.

[49] Health Babies Bright Futures (2017). HBBF Arsenic in 9 Brands of Infant Cereal. https://hbbf.org/report/arsenic-in-9-brands-of-infant-cereal.

[50] Londonio A., Morzán E., Smichowski P. (2019). Determination of Toxic and Potentially Toxic Elements in Rice and Rice-Based Products by Inductively Coupled Plasma-Mass Spectrometry. Food Chem..

[51] Ministry of Health (2017). Resolução da Diretoria Colegiada—RDC. N° 193, de 12 de Dezembro de 2017.

[52] de Souza A.O., Corrêa Pereira C., Heling A.I., Quadro Oreste E., Cadore S., Schwingel Ribeiro A., Antunes Vieira M. (2019). Determination of Total Concentration and Bioaccessible Fraction of Metals in Infant Cereals by MIP OES. J. Food Compos. Anal..

[53] Carbonell-Barrachina Á.A., Ramírez-Gandolfo A., Wu X., Norton G.J., Burló F., Deacon C., Meharg A.A. (2012). Essential and Toxic Elements in Infant Foods from Spain, UK, China and USA. J. Environ. Monit..

[54] JECFA (2011). WHO FOOD ADDITIVES SERIES 64: Safety Evaluation of Certain Food Additives and Contaminants, Prepared by the Seventy-Third Meeting of JECFA. Lead.

[55] EC (2015). Amending Regulation (EC) No 1881/2006 as Regards Maximum Levels of Inorganic Arsenic in Foodstuffs.

[56] EC Commission REGULATION (EU) 2015/1006 of 25 June 2015 Amending Regulation (EC) No 1881/2006 as Regards Maximum Levels of Inorganic Arsenic in Foodstuff. 2015; pp. 14–16. https://eur-lex.europa.eu/eli/reg/2015/1006/oj.

[57] EFSA Panel on Contaminants in the Food Chain (CONTAM) (2011). Statement on Tolerable Weekly Intake for Cadmium. EFSA J..

[58] WHO (2017). Guidelines for Drinking-Water Quality.

[59] Joint FAO/WHO (2017). Code of Practice for the Prevention and Reduction of Arsenic Contamination in Rice.

[60] Toledo M.C., Lee J.S., Batista B.L., Olympio K.P.K., Nardocci A.C. (2022). Exposure to Inorganic Arsenic in Rice in Brazil: A Human Health Risk Assessment. Int. J. Environ. Res. Public Health.

[61] Signes-Pastor A.J., Cottingham K.L., Carey M., Sayarath V., Palys T., Meharg A.A., Folt C.L., Karagas M.R. (2018). Infants’ Dietary Arsenic Exposure during Transition to Solid Food. Sci. Rep..

[62] IARC (2004). Some Drinking-Water Disinfectants and Contaminants, Including Arsenic.

[63] EFSA Panel on Contaminants in the Food Chain (CONTAM) (2009). Scientific Opinion on Arsenic in Food. EFSA J..

[64] Neto M.C.d.V., Silva T.B.C., de Araújo V.E., Souza S.V.C. (2019). de Lead Contamination in Food Consumed and Produced in Brazil: Systematic Review and Meta-Analysis. Food Res. Int..

[65] Leroux I.N., da Silva Ferreira A.P.S., Paniz F.P., Pedron T., Salles F.J., da Silva F.F., Maltez H.F., Batista B.L., Olympio K.P.K. (2018). Lead, Cadmium, and Arsenic Bioaccessibility of 24 h Duplicate Diet Ingested by Preschool Children Attending Day Care Centers in Brazil. Int. J. Environ. Res. Public Health.

[66] Zoroddu M.A., Aaseth J., Crisponi G., Medici S., Peana M., Nurchi V.M. (2019). The Essential Metals for Humans: A Brief Overview. J. Inorg. Biochem..

[67] Sipahi H., Eken A., Aydın A., Şahin G., Baydar T. (2015). Safety Assessment of Essential and Toxic Metals in Infant Formulas. Turk. J. Pediatr..

[68] Ljung K., Palm B., Grandér M., Vahter M. (2011). High Concentrations of Essential and Toxic Elements in Infant Formula and Infant Foods—A Matter of Concern. Food Chem..

[69] ATSDR (2007). Toxicological Profile for Arsenic.

[70] Jean J., Sirot V., Hulin M., Le Calvez E., Zinck J., Noël L., Vasseur P., Nesslany F., Gorecki S., Guérin T. (2018). Dietary Exposure to Cadmium and Health Risk Assessment in Children—Results of the French Infant Total Diet Study. Food Chem. Toxicol..

[71] Olympio K.P.K., Silva J.P.d.R., da Silva A.S., Souza V.C.d.O., Buzalaf M.A.R., Barbosa F., Cardoso M.R.A. (2018). Blood Lead and Cadmium Levels in Preschool Children and Associated Risk Factors in São Paulo, Brazil. Environ. Pollut..

[72] WHO (2003). Global Strategy for Infant and Young Child Feeding.

[73] Horta B.L., Loret De Mola C., Victora C.G. (2015). Long-Term Consequences of Breastfeeding on Cholesterol, Obesity, Systolic Blood Pressure and Type 2 Diabetes: A Systematic Review and Meta-Analysis. Acta Paediatr. Int. J. Paediatr..

[74] Kramer M.S., Guo T., Platt R.W., Vanilovich I., Sevkovskaya Z., Dzikovich I., Michaelsen K.F., Dewey K. (2004). Feeding Effects on Growth during Infancy. J. Pediatr..

[75] JECFA (2011). Safety Evaluation of Certain Contaminants in Food.

[76] U.S. FDA Closer to Zero: Reducing Childhood Exposure to Contaminants from Foods. https://www.fda.gov/food/environmental-contaminants-food/closer-zero-reducing-childhood-exposure-contaminants-foods.

[77] U.S. FDA (2020). Inorganic Arsenic in Rice Cereals for Infants: Action Level Guidance for Industry Table of Contents.

[78] Carey M., Donaldson E., Signes-Pastor A.J., Meharg A.A. (2018). Dilution of Rice with Other Gluten Free Grains to Lower Inorganic Arsenic in Foods for Young Children in Response to European Union Regulations Provides Impetus to Setting Stricter Standards. PLoS ONE.

[79] EC (2016). Scientific Opinion on the Risks to Public Health Related to the Presence of Nickel in Food and Drinking Water.

[80] Albuquerque A.d.O., Dantas K.B., Ariadne M., Gomes Tomé B., Dos J., Aire S., Martins M.C. (2018). Hábitos Alimentares de Crianças Com Até 6 Meses Em Alimentação Complementar e/Ou Desmame Precoce. Rev. Enferm. Atual.

[81] Sombra P.V., Sampaio R.M.M., Silva F.R., Pinto F.J.M. (2017). Alimentação Complementar e Ingestão de Alimentos Industrializados Em Crianças Menores de Três Anos. Saúde E Desenvolv. Hum..

[82] Kato L.S., De Nadai Fernandes E.A., Raab A., Bacchi M.A., Feldmann J. (2019). Arsenic and Cadmium Contents in Brazilian Rice from Different Origins Can Vary More than Two Orders of Magnitude. Food Chem..

[83] Monteiro L.R., Lange C.N., Freire B.M., Pedron T., da Silva J.J.C., de Magalhães A.M., Pegoraro C., Busanello C., Batista B.L. (2020). Inter- and Intra-Variability in the Mineral Content of Rice Varieties Grown in Various Microclimatic Regions of Southern Brazil. J. Food Compos. Anal..

[84] Segura F.R., Franco D.F., da Silva J.J.C., Batista B.L. (2020). Variations in Total As and As Species in Rice Indicate the Need for Crop-Tracking. J. Food Compos. Anal..

[85] Lange C.N., Monteiro L.R., Freire B.M., Franco D.F., de Souza R.O., dos Reis Ferreira C.S., da Silva J.J.C., Batista B.L. (2019). Mineral Profile Exploratory Analysis for Rice Grains Traceability. Food Chem..

[86] WHO (2017). Guideline: Protecting, Promoting and Supporting Breastfeeding in Facilities Providing Maternity and Newborn Services.

[87] WHO (2015). The Global Strategy For Women’s, Children’s and Adolescent’s Health (2016–2030).

[88] (2016). Brasil Constituição da República Federativa do Brasil.

[89] (2009). Brasil Trabalho e Família—Rumo a Novas Formas de Conciliação Com Co-Responsabilidade Social.

[90] Monteiro F.R., Buccini G.d.S., Venâncio S.I., Costa T.H.M. (2017). da Influência Da Licença-Maternidade Sobre a Amamentação Exclusiva. J. Pediatr..

[91] Rimes K.A., de Oliveira M.I.C., Boccolini C.S. (2019). Maternity Leave and Exclusive Breastfeeding. Rev. Saude Publica.

[92] Kamiya E., Mendonça L.A.B.M., Ferreira R.D.S., Palhares D.B. (2019). Prevalência de Aleitamento Materno Em Campo Grande, Mato Grosso Do Sul, Brasil. Multitemas.

[93] Neri V.F., Alves A.L.L., Guimarães L.C. (2019). Prevalência de Desmame Precoce e Fatores Relacionados Em Crianças Do Distrito Federal e Entorno. Rev. Divulg. Científica Sena Aires.

[94] Capucho L.B., Forechi L., Lima R.d.C.D., Massaroni L., Primo C.C. (2017). Fatores Que Interferem Na Amamentação Exclusiva. Rev. Bras. Pesqui. Em Saúde/Brazilian J. Health Res..

[95] Venancio S.I., Giugliani E.R.J., de Oliveira Silva O.L., Stefanello J., Benicio M.H.D., Guerreiro dos Reis M.C., Issler R.M.S., do Espírito Santo L.C., Cardoso M.R.A., Rios G.S. (2016). Associação Entre o Grau de Implantação Da Rede Amamenta Brasil e Indicadores de Amamentação. Cad. Saude Publica.

[96] Rito R.V.V.F., De Oliveira M.I.C., Brito A.D.S. (2013). Degree of Compliance with the Ten Steps of the Breastfeeding Friendly Primary Care Initiative and Its Association with the Prevalence of Exclusive Breastfeeding. J. Pediatr..

[97] Garcia C.F., Orcid H.J.V. (2018). Implicações Do Retorno Ao Trabalho Após Licença-Maternidade Na Rotina e No Trabalho Da Mulher Implications of Returning to Work after Maternity Leave in Routine and in Women’s Work. Fractal Rev. Psicol..

[98] Ministry of Health (2015). Cartilha Para Mulher Trabalhadora Que Amamenta.

[99] Ministry of Health (2010). Portaria N° 193, de 23 de Fevereiro de 2010. Discorre Sobre a Instalação de Salas de Apoio à Amamentação Em Empresas Públicas Ou Privadas, e a Fiscalização Desses Ambientes Pelas Vigilâncias Sanitárias Locais.

[100] Brasil (1943). Decreto-Lei N° 452, de 1° de Maio de 1943. Aprova a Consolidação Das Leis Do Trabalho. Diario Oficial Uniao. 9 Ago 1943.

[101] Brasil (2008). Lei n 11.770 de 9 de Setembro de 2008.

[102] São Paulo (2011). Manual de Orientação Para Centros de Educação Infantil—CEI. Esquema Alimentar e Porcionamentos.

