# Supplementary material for: Essential and Toxic Elements in Infant Cereal in Brazil: Exposure Risk Assessment
Source: Int J Environ Res Public Health. 2024 Mar 22;21(4):381. doi: 10.3390/ijerph21040381 (PMC11050093; doi:10.3390/ijerph21040381)
Supplement: Supplementary file 1 [file ijerph-21-00381-s001.zip › ijerph-2899230-supplementary.pdf]

Supplementary material

Table S1
Estimated incremental cancer risks and incremental lifetime cancer risks associated with exposure to non-essential elements present in different kinds of infant cereal.

Type of infant cereal (ID)	Age group	iAs	Pb	Total
Corn (A)	4 to < 12 months	2.72×10^{-07}	2.78×10^{-09}	2.74×10^{-07}
	12 to < 24 months	8.55×10^{-07}	8.76×10^{-09}	8.64×10^{-07}
	**ILCR**	**1.13×10^{-06}**	**1.15×10^{-08}**	**1.14×10^{-06}**
Oatmeal (B)	4 to < 12 months	7.16×10^{-07}	8.63×10^{-09}	7.25×10^{-07}
	12 to < 24 months	2.26×10^{-06}	2.72×10^{-08}	2.28×10^{-06}
	**ILCR**	**2.97×10^{-06}**	**3.58×10^{-08}**	**3.01×10^{-06}**
Oatmeal (C)	4 to < 12 months	3.60×10^{-07}	5.38×10^{-09}	3.65×10^{-07}
	12 to < 24 months	1.13×10^{-06}	1.70×10^{-08}	1.15×10^{-06}
	**ILCR**	**1.49×10^{-06}**	**2.23×10^{-08}**	**1.52×10^{-06}**
Multi-grain (D)	4 to < 12 months	7.35×10^{-07}	1.01×10^{-08}	7.45×10^{-07}
	12 to < 24 months	2.31×10^{-06}	3.19×10^{-08}	2.35×10^{-06}
	**ILCR**	**3.05×10^{-06}**	**4.21×10^{-08}**	**3.09×10^{-06}**
Rice and oat (E)	4 to < 12 months	1.50×10^{-06}	1.95×10^{-08}	1.52×10^{-06}
	12 to < 24 months	4.74×10^{-06}	6.13×10^{-08}	4.80×10^{-06}
	**ILCR**	**6.24×10^{-06}**	**8.08×10^{-08}**	**6.32×10^{-06}**
Rice and oat (F)	4 to < 12 months	7.01×10^{-06}	8.26×10^{-09}	7.02×10^{-06}
	12 to < 24 months	2.21×10^{-05}	2.60×10^{-08}	2.21×10^{-05}
	**ILCR**	**2.91×10^{-05}**	**3.43×10^{-08}**	**2.91×10^{-05}**
Rice and fruit (G)	4 to < 12 months	5.20×10^{-06}	9.08×10^{-09}	5.21×10^{-06}
	12 to < 24 months	1.64×10^{-05}	2.86×10^{-08}	1.64×10^{-05}
	**ILCR**	**2.16×10^{-05}**	**3.77×10^{-08}**	**2.16×10^{-05}**
Rice and cornstarch (H)	4 to < 12 months	1.08×10^{-06}	7.31×10^{-09}	1.09×10^{-06}
	12 to < 24 months	3.41×10^{-06}	2.30×10^{-08}	3.43×10^{-06}
	**ILCR**	**4.49×10^{-06}**	**3.03×10^{-08}**	**4.52×10^{-06}**
Rice and cornstarch (I)	4 to < 12 months	9.74×10^{-07}	6.56×10^{-09}	9.81×10^{-07}
	12 to < 24 months	3.07×10^{-06}	2.07×10^{-08}	3.09×10^{-06}
	**ILCR**	**4.04×10^{-06}**	**2.72×10^{-08}**	**4.07×10^{-06}**
Rice (J)	4 to < 12 months	6.48×10^{-06}	1.62×10^{-08}	6.50×10^{-06}
	12 to < 24 months	2.04×10^{-05}	5.10×10^{-08}	2.05×10^{-05}
	**ILCR**	**2.69×10^{-05}**	**6.72×10^{-08}**	**2.70×10^{-05}**
Rice (K)	4 to < 12 months	6.49×10^{-06}	8.00×10^{-09}	6.50×10^{-06}
	12 to < 24 months	2.05×10^{-05}	2.52×10^{-08}	2.05×10^{-05}
	**ILCR**	**2.70×10^{-05}**	**3.32×10^{-08}**	**2.70×10^{-05}**
Rice (L)	4 to < 12 months	7.18×10^{-06}	1.04×10^{-08}	7.19×10^{-06}
	12 to < 24 months	2.26×10^{-05}	3.27×10^{-08}	2.27×10^{-05}
	**ILCR**	**2.98×10^{-05}**	**4.31×10^{-08}**	**2.99×10^{-05}**
Rice (M)	4 to < 12 months	8.36×10^{-06}	6.64×10^{-09}	8.36×10^{-06}
	12 to < 24 months	2.63×10^{-05}	2.09×10^{-08}	2.63×10^{-05}
	**ILCR**	**3.47×10^{-05}**	**2.76×10^{-08}**	**3.47×10^{-05}**
Rice (N)	4 to < 12 months	5.85×10^{-06}	7.34×10^{-09}	5.86×10^{-06}
	12 to < 24 months	1.84×10^{-05}	2.31×10^{-08}	1.84×10^{-05}
	**ILCR**	**2.43×10^{-05}**	**3.05×10^{-08}**	**2.43×10^{-05}**
Rice (O)	4 to < 12 months	9.60×10^{-06}	5.04×10^{-09}	9.61×10^{-06}
	12 to < 24 months	3.03×10^{-05}	1.59×10^{-08}	3.03×10^{-05}

	**ILCR**	**3.99×10^{-05}**	**2.09×10^{-08}**	**3.99×10^{-05}**
Rice (P)	4 to < 12 months	4.95×10^{-06}	5.30×10^{-09}	4.96×10^{-06}
	12 to < 24 months	1.56×10^{-05}	1.67×10^{-08}	1.56×10^{-05}
	**ILCR**	**2.06×10^{-05}**	**2.20×10^{-08}**	**2.06×10^{-05}**
Rice (Q)	4 to < 12 months	7.48×10^{-06}	1.07×10^{-08}	7.49×10^{-06}
	12 to < 24 months	2.36×10^{-05}	3.38×10^{-08}	2.36×10^{-05}
	**ILCR**	**3.10×10^{-05}**	**4.46×10^{-08}**	**3.11×10^{-05}**
Rice (R)	4 to < 12 months	8.73×10^{-07}	1.01×10^{-08}	8.83×10^{-07}
	12 to < 24 months	2.75×10^{-06}	3.19×10^{-08}	2.78×10^{-06}
	**ILCR**	**3.62×10^{-06}**	**4.20×10^{-08}**	**3.66×10^{-06}**

iAs = inorganic arsenic; ILCR = incremental lifetime cancer risk; Pb = lead.

Table S2

Estimated average daily dose associated with exposure to essential and non-essential elements present in different kinds of infant cereal.

Type of infant cereal (ID)	Age group	Essential elements (mg/kg per day)											Non-essential elements (mg/kg per day)			
		Ag	B	Ba	Co	Cr	Cu	Mn	Ni	Se	Sr	Zn	As	Al	Cd	Pb
Corn (A)	4 to < 12 months	2.00×10^{-10}	2.00×10^{-10}	1.23×10^{-03}	2.58×10^{-05}	4.37×10^{-04}	4.85×10^{-03}	9.93×10^{-03}	3.09×10^{-04}	2.48×10^{-04}	2.48×10^{-02}	3.08×10^{-01}	1.90×10^{-05}	9.04×10^{-03}	3.07×10^{-06}	3.43×10^{-05}
	12 to < 24 months	4.20×10^{-10}	4.20×10^{-10}	2.59×10^{-03}	5.42×10^{-05}	9.19×10^{-04}	1.02×10^{-02}	2.08×10^{-02}	6.48×10^{-04}	5.21×10^{-04}	5.20×10^{-02}	6.47×10^{-01}	3.99×10^{-05}	1.90×10^{-02}	6.44×10^{-06}	7.21×10^{-05}
Oatmeal (B)	4 to < 12 months	9.87×10^{-05}	5.40×10^{-04}	1.29×10^{-02}	1.18×10^{-04}	3.60×10^{-04}	1.07×10^{-02}	1.04×10^{-01}	1.99×10^{-03}	2.73×10^{-05}	2.94×10^{-02}	3.71×10^{-01}	5.01×10^{-05}	7.01×10^{-03}	6.33×10^{-06}	1.07×10^{-04}
	12 to < 24 months	2.07×10^{-04}	1.13×10^{-03}	2.71×10^{-02}	2.48×10^{-04}	7.57×10^{-04}	2.24×10^{-02}	2.18×10^{-01}	4.19×10^{-03}	5.74×10^{-05}	6.17×10^{-02}	7.80×10^{-01}	1.05×10^{-04}	1.47×10^{-02}	1.33×10^{-05}	2.24×10^{-04}
Oatmeal (C)	4 to < 12 months	2.00×10^{-10}	2.00×10^{-10}	9.26×10^{-03}	4.68×10^{-05}	3.30×10^{-04}	1.36×10^{-02}	1.12×10^{-01}	1.67×10^{-03}	2.13×10^{-04}	2.74×10^{-02}	3.52×10^{-01}	2.52×10^{-05}	1.41×10^{-02}	7.40×10^{-06}	6.65×10^{-05}
	12 to < 24 months	4.20×10^{-10}	4.20×10^{-10}	1.94×10^{-02}	9.82×10^{-05}	6.93×10^{-04}	2.85×10^{-02}	2.35×10^{-01}	3.50×10^{-03}	4.48×10^{-04}	5.76×10^{-02}	7.39×10^{-01}	5.29×10^{-05}	2.95×10^{-02}	1.55×10^{-05}	1.40×10^{-04}
Multi-grain (D)	4 to < 12 months	2.00×10^{-10}	2.00×10^{-10}	5.08×10^{-03}	4.65×10^{-05}	4.72×10^{-04}	6.93×10^{-03}	3.23×10^{-02}	3.29×10^{-04}	1.48×10^{-04}	3.97×10^{-03}	5.32×10^{-01}	5.14×10^{-05}	1.12×10^{-02}	3.81×10^{-05}	1.25×10^{-04}
	12 to < 24 months	4.20×10^{-10}	4.20×10^{-10}	1.07×10^{-02}	9.76×10^{-05}	9.91×10^{-04}	1.45×10^{-02}	6.78×10^{-02}	6.91×10^{-04}	3.11×10^{-04}	8.34×10^{-03}	1.12×10^{00}	1.08×10^{-04}	2.35×10^{-02}	8.00×10^{-05}	2.63×10^{-04}
Rice and oat (E)	4 to < 12 months	4.19×10^{-05}	3.28×10^{-03}	2.32×10^{-03}	5.79×10^{-05}	1.36×10^{-03}	1.18×10^{-02}	6.45×10^{-02}	1.67×10^{-03}	3.02×10^{-03}	3.19×10^{-05}	1.63×10^{-01}	1.05×10^{-04}	3.52×10^{-02}	1.37×10^{-05}	2.41×10^{-04}
	12 to < 24 months	8.79×10^{-05}	6.89×10^{-03}	4.87×10^{-03}	1.22×10^{-04}	2.85×10^{-03}	2.47×10^{-02}	1.35×10^{-01}	3.50×10^{-03}	6.33×10^{-03}	6.71×10^{-05}	3.43×10^{-01}	2.21×10^{-04}	7.39×10^{-02}	2.88×10^{-05}	5.05×10^{-04}
Rice and oat (F)	4 to < 12 months	4.72×10^{-05}	5.71×10^{-04}	4.41×10^{-03}	7.94×10^{-05}	1.98×10^{-04}	6.34×10^{-03}	4.14×10^{-02}	7.29×10^{-04}	1.24×10^{-04}	1.91×10^{-01}	2.67×10^{-01}	4.91×10^{-04}	1.55×10^{-02}	5.19×10^{-05}	1.02×10^{-04}
	12 to < 24 months	9.91×10^{-05}	1.20×10^{-03}	9.25×10^{-03}	1.67×10^{-04}	4.16×10^{-04}	1.33×10^{-02}	8.69×10^{-02}	1.53×10^{-03}	2.61×10^{-04}	4.02×10^{-01}	5.61×10^{-01}	1.03×10^{-03}	3.25×10^{-02}	1.09×10^{-04}	2.14×10^{-04}
Rice and fruit (G)	4 to < 12 months	1.29×10^{-04}	7.63×10^{-03}	4.85×10^{-03}	2.45×10^{-04}	8.38×10^{-04}	1.69×10^{-02}	5.29×10^{-02}	1.43×10^{-03}	1.80×10^{-04}	1.30×10^{-02}	6.40×10^{-02}	3.64×10^{-04}	1.89×10^{-02}	5.71×10^{-05}	1.12×10^{-04}
	12 to < 24 months	2.70×10^{-04}	1.60×10^{-02}	1.02×10^{-02}	5.14×10^{-04}	1.76×10^{-03}	3.55×10^{-02}	1.11×10^{-01}	3.01×10^{-03}	3.79×10^{-04}	2.73×10^{-02}	1.34×10^{-01}	7.64×10^{-04}	3.97×10^{-02}	1.20×10^{-04}	2.36×10^{-04}
Rice and cornstarch (H)	4 to < 12 months	2.00×10^{-10}	2.00×10^{-10}	1.66×10^{-03}	5.79×10^{-05}	1.53×10^{-03}	1.25×10^{-02}	5.28×10^{-02}	1.94×10^{-03}	1.94×10^{-04}	6.61×10^{-02}	2.69×10^{-01}	7.57×10^{-05}	1.87×10^{-02}	1.73×10^{-05}	9.03×10^{-05}
	12 to < 24 months	4.20×10^{-10}	4.20×10^{-10}	3.48×10^{-03}	1.21×10^{-04}	3.22×10^{-03}	2.62×10^{-02}	1.11×10^{-01}	4.07×10^{-03}	4.08×10^{-04}	1.39×10^{-01}	5.64×10^{-01}	1.59×10^{-04}	3.92×10^{-02}	3.64×10^{-05}	1.90×10^{-04}
Rice and cornstarch (I)	4 to < 12 months	2.00×10^{-10}	2.00×10^{-10}	1.42×10^{-05}	2.12×10^{-05}	5.56×10^{-04}	8.10×10^{-04}	4.09×10^{-03}	3.23×10^{-04}	1.90×10^{-04}	3.41×10^{-04}	4.35×10^{-01}	6.82×10^{-05}	5.06×10^{-03}	1.22×10^{-05}	8.10×10^{-05}
	12 to <24 months	4.20×10^{-10}	4.20×10^{-10}	2.99×10^{-05}	4.45×10^{-05}	1.17×10^{-03}	1.70×10^{-03}	8.59×10^{-03}	6.77×10^{-04}	3.99×10^{-04}	7.15×10^{-04}	9.14×10^{-01}	1.43×10^{-04}	1.06×10^{-02}	2.57×10^{-05}	1.70×10^{-04}
Rice (J)	4 to < 12 months	2.00×10^{-10}	2.00×10^{-10}	1.21×10^{-03}	4.77×10^{-05}	3.75×10^{-04}	6.92×10^{-03}	2.81×10^{-02}	6.93×10^{-04}	1.08×10^{-04}	1.86×10^{-02}	2.79×10^{-01}	4.54×10^{-04}	9.64×10^{-03}	8.26×10^{-06}	2.00×10^{-04}
	12 to < 24 months	4.20×10^{-10}	4.20×10^{-10}	2.55×10^{-03}	1.00×10^{-04}	7.88×10^{-04}	1.45×10^{-02}	5.90×10^{-02}	1.46×10^{-03}	2.26×10^{-04}	3.91×10^{-02}	5.86×10^{-01}	9.53×10^{-04}	2.02×10^{-02}	1.74×10^{-05}	4.20×10^{-04}
Rice (K)	4 to < 12 months	8.65×10^{-05}	2.00×10^{-10}	1.35×10^{-03}	9.34×10^{-05}	2.86×10^{-04}	8.21×10^{-03}	4.12×10^{-02}	8.83×10^{-04}	4.01×10^{-03}	1.30×10^{-04}	4.86×10^{-01}	4.55×10^{-04}	2.00×10^{-10}	6.86×10^{-05}	9.88×10^{-05}
	12 to < 24 months	1.82×10^{-04}	4.20×10^{-10}	2.83×10^{-03}	1.96×10^{-04}	6.00×10^{-04}	1.72×10^{-02}	8.66×10^{-02}	1.85×10^{-03}	8.41×10^{-03}	2.72×10^{-04}	1.02×10^{00}	9.55×10^{-04}	4.20×10^{-10}	1.44×10^{-04}	2.08×10^{-04}
Rice (L)	4 to < 12 months	2.00×10^{-10}	2.00×10^{-10}	8.58×10^{-04}	1.11×10^{-04}	2.42×10^{-04}	8.79×10^{-03}	4.59×10^{-02}	6.88×10^{-04}	1.31×10^{-04}	3.10×10^{-03}	5.07×10^{-01}	5.03×10^{-04}	2.21×10^{-03}	7.65×10^{-05}	1.28×10^{-04}
	12 to <24 months	4.20×10^{-10}	4.20×10^{-10}	1.80×10^{-03}	2.34×10^{-04}	5.07×10^{-04}	1.85×10^{-02}	9.65×10^{-02}	1.45×10^{-03}	2.76×10^{-04}	6.51×10^{-03}	1.07×10^{00}	1.06×10^{-03}	4.64×10^{-03}	1.61×10^{-04}	2.69×10^{-04}
Rice (M)	4 to < 12 months	1.32×10^{-04}	1.24×10^{-03}	2.27×10^{-03}	7.06×10^{-05}	1.07×10^{-03}	7.55×10^{-03}	5.64×10^{-02}	7.33×10^{-04}	1.12×10^{-04}	4.51×10^{-03}	4.98×10^{-01}	5.85×10^{-04}	3.25×10^{-04}	8.86×10^{-05}	8.21×10^{-05}
	12 to < 24 months	2.76×10^{-04}	2.61×10^{-03}	4.76×10^{-03}	1.48×10^{-04}	2.24×10^{-03}	1.59×10^{-02}	1.18×10^{-01}	1.54×10^{-03}	2.36×10^{-04}	9.48×10^{-03}	1.05×10^{00}	1.23×10^{-03}	6.83×10^{-04}	1.86×10^{-04}	1.72×10^{-04}

Rice (N)	4 to < 12 months	1.10×10^{-04}	4.25×10^{-03}	5.92×10^{-04}	9.82×10^{-05}	2.80×10^{-04}	7.82×10^{-03}	3.63×10^{-02}	4.51×10^{-04}	1.91×10^{-04}	4.93×10^{-04}	4.59×10^{-01}	4.09×10^{-04}	2.00×10^{-10}	5.55×10^{-05}	9.07×10^{-05}
	12 to < 24 months	2.30×10^{-04}	8.92×10^{-03}	1.24×10^{-03}	2.06×10^{-04}	5.88×10^{-04}	1.64×10^{-02}	7.63×10^{-02}	9.47×10^{-04}	4.02×10^{-04}	1.04×10^{-03}	9.64×10^{-01}	8.60×10^{-04}	4.20×10^{-10}	1.17×10^{-04}	1.90×10^{-04}
Rice (O)	4 to < 12 months	2.59×10^{-04}	2.00×10^{-10}	1.69×10^{-03}	2.99×10^{-04}	7.21×10^{-04}	1.17×10^{-02}	6.05×10^{-02}	1.06×10^{-03}	2.14×10^{-04}	7.09×10^{-04}	4.28×10^{-01}	6.72×10^{-04}	3.46×10^{-03}	7.48×10^{-05}	6.23×10^{-05}
	12 to < 24 months	5.43×10^{-04}	4.20×10^{-10}	3.55×10^{-03}	6.28×10^{-04}	1.51×10^{-03}	2.46×10^{-02}	1.27×10^{-01}	2.23×10^{-03}	4.49×10^{-04}	1.49×10^{-03}	8.98×10^{-01}	1.41×10^{-03}	7.27×10^{-03}	1.57×10^{-04}	1.31×10^{-04}
Rice (P)	4 to < 12 months	6.80×10^{-05}	6.29×10^{-03}	4.77×10^{-03}	2.18×10^{-04}	8.47×10^{-04}	1.72×10^{-02}	5.77×10^{-02}	1.73×10^{-03}	1.71×10^{-04}	1.58×10^{-02}	2.31×10^{-01}	3.47×10^{-04}	1.98×10^{-02}	4.34×10^{-05}	6.55×10^{-05}
	12 to < 24 months	1.43×10^{-04}	1.32×10^{-02}	1.00×10^{-02}	4.58×10^{-04}	1.78×10^{-03}	3.61×10^{-02}	1.21×10^{-01}	3.64×10^{-03}	3.59×10^{-04}	3.33×10^{-02}	4.85×10^{-01}	7.28×10^{-04}	4.17×10^{-02}	9.12×10^{-05}	1.38×10^{-04}
Rice (Q)	4 to < 12 months	1.94×10^{-04}	2.00×10^{-10}	1.77×10^{-03}	1.33×10^{-04}	1.53×10^{-04}	1.15×10^{-02}	3.98×10^{-02}	8.60×10^{-04}	5.42×10^{-05}	2.77×10^{-02}	2.14×10^{-01}	5.23×10^{-04}	1.42×10^{-02}	5.01×10^{-05}	1.33×10^{-04}
	12 to < 24 months	4.08×10^{-04}	4.20×10^{-10}	3.71×10^{-03}	2.79×10^{-04}	3.22×10^{-04}	2.41×10^{-02}	8.36×10^{-02}	1.81×10^{-03}	1.14×10^{-04}	5.82×10^{-02}	4.49×10^{-01}	1.10×10^{-03}	2.98×10^{-02}	1.05×10^{-04}	2.79×10^{-04}
Rice (R)	4 to < 12 months	4.38×10^{-06}	2.00×10^{-10}	2.25×10^{-04}	5.92×10^{-05}	8.45×10^{-04}	1.34×10^{-03}	6.47×10^{-03}	3.12×10^{-04}	3.31×10^{-03}	2.09×10^{-05}	5.21×10^{-01}	6.11×10^{-05}	2.00×10^{-10}	9.17×10^{-06}	1.25×10^{-04}
	12 to < 24 months	9.20×10^{-06}	4.20×10^{-10}	4.72×10^{-04}	1.24×10^{-04}	1.77×10^{-03}	2.81×10^{-03}	1.36×10^{-02}	6.55×10^{-04}	6.95×10^{-03}	4.38×10^{-05}	1.09×10^{00}	1.28×10^{-04}	4.20×10^{-10}	1.93×10^{-05}	2.62×10^{-04}

Ag = silver; Al = aluminum; As = arsenic; B = boron; Ba = barium; Cd = cadmium; Co = cobalt; Cr = chromium; Cu = copper; Mn = manganese; Ni = nickel; Pb = lead; Se = selenium; Sr = strontium; Zn = zinc..

Table S3

Estimated incremental lifetime cancer risk associated with exposure to non-essential elements present in different kinds of infant cereal, if inorganic arsenic is assumed to account for 52% of the total arsenic.

Type of infant cereal (ID)	iAs	Pb	Total
Corn (A)	5.86×10^{-07}	1.15×10^{-08}	6.31×10^{-07}
Oatmeal (B)	1.55×10^{-06}	3.58×10^{-08}	1.67×10^{-06}
Oatmeal (C)	7.76×10^{-07}	2.23×10^{-08}	8.43×10^{-07}
Multi-grain (D)	1.59×10^{-06}	4.21×10^{-08}	1.72×10^{-06}
Rice and oat (E)	3.25×10^{-06}	8.08×10^{-08}	3.51×10^{-06}
Rice and oat (F)	1.51×10^{-05}	3.43×10^{-08}	1.60×10^{-05}
Rice and fruit (G)	1.12×10^{-05}	3.77×10^{-08}	1.19×10^{-05}
Rice and cornstarch (H)	2.33×10^{-06}	3.03×10^{-08}	2.50×10^{-06}
Rice and cornstarch (I)	2.10×10^{-06}	2.72×10^{-08}	2.25×10^{-06}
Rice (J)	1.40×10^{-05}	6.72×10^{-08}	1.49×10^{-05}
Rice (K)	1.40×10^{-05}	3.32×10^{-08}	1.49×10^{-05}
Rice (L)	1.55×10^{-05}	4.31×10^{-08}	1.64×10^{-05}
Rice (M)	1.80×10^{-05}	2.76×10^{-08}	1.91×10^{-05}
Rice (N)	1.26×10^{-05}	3.05×10^{-08}	1.34×10^{-05}
Rice (O)	2.07×10^{-05}	2.09×10^{-08}	2.19×10^{-05}
Rice (P)	1.07×10^{-05}	2.20×10^{-08}	1.13×10^{-05}
Rice (Q)	1.61×10^{-05}	4.46×10^{-08}	1.71×10^{-05}
Rice (R)	1.88×10^{-06}	4.20×10^{-08}	2.03×10^{-06}

iAs = inorganic arsenic; Pb = lead.

Table S4

Fractional and lifetime hazard quotients* for exposure to essential and non-essential elements present in infant cereal.

		Essential elements											Non-essential elements			
**Type of infant cereal (ID)**	**Age group**	**Ag**	**B**	**Ba**	**Co**	**Cr**	**Cu**	**Mn**	**Ni**	**Se**	**Sr**	**Zn**	**As**	**Al**	**Cd**	**Pb**
Corn (A)	4 to <12 months	1.60×10^{-08}	4.00×10^{-10}	2.5×10^{-03}	1.0×10^{-03}	1.2×10^{-04}	1.9×10^{-01}	1.3×10^{-01}	1.1×10^{-02}	2.0×10^{-02}	1.7×10^{-02}	4.1×10^{-01}	2.5×10^{-02}	3.6×10^{-03}	1.1×10^{-01}	3.9×10^{-03}
	12 to < 24 months	5.04×10^{-08}	1.26×10^{-09}	7.8×10^{-03}	3.3×10^{-03}	3.7×10^{-04}	6.1×10^{-01}	4.2×10^{-01}	3.5×10^{-02}	6.3×10^{-02}	5.2×10^{-02}	1.3×10^{00}	8.0×10^{-02}	1.1×10^{-02}	3.5×10^{-01}	1.2×10^{-02}
	**4 to < 24 months**	**6.64×10^{-08}**	1.66×10^{-09}	**1.0×10^{-02}**	**4.3×10^{-03}**	**4.8×10^{-04}**	**8.1×10^{-01}**	**5.5×10^{-01}**	**4.7×10^{-02}**	**8.2×10^{-02}**	**6.9×10^{-02}**	**1.7×10^{00}**	**1.1×10^{-01}**	**1.5×10^{-02}**	**4.6×10^{-01}**	**1.6×10^{-02}**
Oatmeal (B)	0.5 to 1 year	7.90×10^{-03}	1.08×10^{-03}	2.6×10^{-02}	4.7×10^{-03}	9.6×10^{-05}	4.3×10^{-01}	1.4×10^{00}	7.3×10^{-02}	2.2×10^{-03}	2.0×10^{-02}	5.0×10^{-01}	6.7×10^{-02}	2.8×10^{-03}	2.3×10^{-01}	1.2×10^{-02}
	1 to 2 years	2.49×10^{-02}	3.40×10^{-03}	8.1×10^{-02}	1.5×10^{-02}	3.0×10^{-04}	1.3×10^{00}	4.4×10^{00}	2.3×10^{-01}	6.9×10^{-03}	6.2×10^{-02}	1.6×10^{00}	2.1×10^{-01}	8.8×10^{-03}	7.2×10^{-01}	3.8×10^{-02}
	**4 to < 24 months**	**3.28×10^{-02}**	4.48×10^{-03}	**1.1×10^{-01}**	**2.0×10^{-02}**	**4.0×10^{-04}**	**1.8×10^{00}**	**5.7×10^{00}**	**3.0×10^{-01}**	**9.1×10^{-03}**	**8.1×10^{-02}**	**2.1×10^{00}**	**2.8×10^{-01}**	**1.2×10^{-02}**	**9.5×10^{-01}**	**5.1×10^{-02}**
Oatmeal (C)	0.5 to 1 year	1.60×10^{-08}	4.00×10^{-10}	1.9×10^{-02}	1.9×10^{-03}	8.8×10^{-05}	5.4×10^{-01}	1.5×10^{00}	6.1×10^{-02}	1.7×10^{-02}	1.8×10^{-02}	4.7×10^{-01}	3.4×10^{-02}	5.6×10^{-03}	2.7×10^{-01}	7.6×10^{-03}
	1 to 2 years	5.04×10^{-08}	1.26×10^{-09}	5.8×10^{-02}	5.9×10^{-03}	2.8×10^{-04}	1.7×10^{00}	4.7×10^{00}	1.9×10^{-01}	5.4×10^{-02}	5.8×10^{-02}	1.5×10^{00}	1.1×10^{-01}	1.8×10^{-02}	8.5×10^{-01}	2.4×10^{-02}
	**4 to < 24 months**	**6.64×10^{-08}**	1.66×10^{-09}	**7.7×10^{-02}**	**7.8×10^{-03}**	**3.7×10^{-04}**	**2.3×10^{00}**	**6.2×10^{00}**	**2.5×10^{-01}**	**7.1×10^{-02}**	**7.6×10^{-02}**	**1.9×10^{00}**	**1.4×10^{-01}**	**2.3×10^{-02}**	**1.1×10^{00}**	**3.2×10^{-02}**
Multi-grain (D)	0.5 to 1 year	1.60×10^{-08}	4.00×10^{-10}	1.0×10^{-02}	1.9×10^{-03}	1.3×10^{-04}	2.8×10^{-01}	4.3×10^{-01}	1.2×10^{-02}	1.2×10^{-02}	2.6×10^{-03}	7.1×10^{-01}	6.9×10^{-02}	4.48×10^{-03}	1.4×10^{00}	1.4×10^{-02}
	1 to 2 years	5.04×10^{-08}	1.26×10^{-09}	3.2×10^{-02}	5.9×10^{-03}	4.0×10^{-04}	8.7×10^{-01}	1.4×10^{00}	3.8×10^{-02}	3.7×10^{-02}	8.3×10^{-03}	2.2×10^{00}	2.2×10^{-01}	1.41×10^{-02}	4.4×10^{00}	4.5×10^{-02}
	**4 to < 24 months**	**6.64×10^{-08}**	1.66×10^{-09}	**4.2×10^{-02}**	**7.7×10^{-03}**	**5.2×10^{-04}**	**1.1×10^{00}**	**1.8×10^{00}**	**5.0×10^{-02}**	**4.9×10^{-02}**	**1.1×10^{-02}**	**2.9×10^{00}**	**2.8×10^{-01}**	**1.86×10^{-02}**	**5.7×10^{00}**	**5.9×10^{-02}**
Rice and oat (E)	0.5 to 1 year	3.35×10^{-03}	6.57×10^{-03}	4.6×10^{-03}	2.3×10^{-03}	3.6×10^{-04}	4.7×10^{-01}	8.6×10^{-01}	6.1×10^{-02}	2.4×10^{-01}	2.1×10^{-05}	2.2×10^{-01}	1.4×10^{-01}	1.41×10^{-02}	5.0×10^{-01}	2.7×10^{-02}
	1 to 2 years	1.05×10^{-02}	2.07×10^{-02}	1.5×10^{-02}	7.3×10^{-03}	1.1×10^{-03}	1.5×10^{00}	2.7×10^{00}	1.9×10^{-01}	7.6×10^{-01}	6.7×10^{-05}	6.9×10^{-01}	4.4×10^{-01}	4.44×10^{-02}	1.6×10^{00}	8.7×10^{-02}
	**4 to < 24 months**	**1.39×10^{-02}**	2.73×10^{-02}	**1.9×10^{-02}**	**9.6×10^{-03}**	**1.5×10^{-03}**	**2.0×10^{00}**	**3.6×10^{00}**	**2.5×10^{-01}**	**1.0×10^{00}**	**8.8×10^{-05}**	**9.0×10^{-01}**	**5.8×10^{-01}**	**5.84×10^{-02}**	**2.1×10^{00}**	**1.1×10^{-01}**
Rice and oat (F)	0.5 to 1 year	3.77×10^{-03}	1.14×10^{-03}	8.81×10^{-03}	3.18×10^{-03}	5.3×10^{-05}	2.5×10^{-01}	5.5×10^{-01}	2.6×10^{-02}	9.9×10^{-03}	1.3×10^{-01}	3.6×10^{-01}	6.5×10^{-01}	6.19×10^{-03}	1.9×10^{00}	1.2×10^{-02}
	1 to 2 years	1.19×10^{-02}	3.59×10^{-03}	2.78×10^{-02}	1.00×10^{-02}	1.7×10^{-04}	8.0×10^{-01}	1.7×10^{00}	8.3×10^{-02}	3.1×10^{-02}	4.0×10^{-01}	1.1×10^{00}	2.1×10^{00}	2.0×10^{-02}	5.9×10^{00}	3.7×10^{-02}
	**4 to < 24 months**	**1.57×10^{-02}**	4.74×10^{-03}	**3.66×10^{-02}**	**1.32×10^{-02}**	**2.2×10^{-04}**	**1.1×10^{00}**	**2.3×10^{00}**	**1.1×10^{-01}**	**4.1×10^{-02}**	**5.3×10^{-01}**	**1.5×10^{00}**	**2.7×10^{00}**	**2.6×10^{-02}**	**7.8×10^{00}**	**4.8×10^{-02}**
Rice and fruit (G)	0.5 to 1 year	1.03×10^{-02}	1.53×10^{-02}	9.70×10^{-03}	9.79×10^{-03}	2.2×10^{-04}	6.8×10^{-01}	7.1×10^{-01}	5.2×10^{-02}	1.4×10^{-02}	8.7×10^{-03}	8.5×10^{-02}	4.9×10^{-01}	7.6×10^{-03}	2.1×10^{00}	1.3×10^{-02}
	1 to 2 years	3.24×10^{-02}	4.81×10^{-02}	3.05×10^{-02}	3.09×10^{-02}	7.0×10^{-04}	2.1×10^{00}	2.2×10^{00}	1.6×10^{-01}	4.5×10^{-02}	2.7×10^{-02}	2.7×10^{-01}	1.5×10^{00}	2.4×10^{-02}	6.5×10^{00}	4.0×10^{-02}
	**4 to < 24 months**	**4.27×10^{-02}**	6.34×10^{-02}	**4.02×10^{-02}**	**4.06×10^{-02}**	**9.3×10^{-04}**	**2.8×10^{00}**	**2.9×10^{00}**	**2.2×10^{-01}**	**6.0×10^{-02}**	**3.6×10^{-02}**	**3.5×10^{-01}**	**2.0×10^{00}**	**3.1×10^{-02}**	**8.6×10^{00}**	**5.3×10^{-02}**
Rice and cornstarch (H)	0.5 to 1 year	1.60×10^{-08}	4.00×10^{-10}	3.32×10^{-03}	2.31×10^{-03}	4.1×10^{-04}	5.0×10^{-01}	7.0×10^{-01}	7.0×10^{-02}	1.6×10^{-02}	4.4×10^{-02}	3.6×10^{-01}	1.0×10^{-01}	7.5×10^{-03}	6.3×10^{-01}	1.0×10^{-02}
	1 to 2 years	5.04×10^{-08}	1.26×10^{-09}	1.04×10^{-02}	7.29×10^{-03}	1.3×10^{-03}	1.6×10^{00}	2.2×10^{00}	2.2×10^{-01}	4.9×10^{-02}	1.4×10^{-01}	1.1×10^{00}	3.2×10^{-01}	2.4×10^{-02}	2.0×10^{00}	3.3×10^{-02}
	**4 to < 24 months**	**6.64×10^{-08}**	1.66×10^{-09}	**1.38×10^{-02}**	**9.60×10^{-03}**	**1.7×10^{-03}**	**2.1×10^{00}**	**2.9×10^{00}**	**2.9×10^{-01}**	**6.5×10^{-02}**	**1.8×10^{-01}**	**1.5×10^{00}**	**4.2×10^{-01}**	**3.1×10^{-02}**	**2.6×10^{00}**	**4.3×10^{-02}**
Rice and cornstarch (I)	0.5 to 1 year	1.60×10^{-08}	4.00×10^{-10}	2.85×10^{-05}	8.48×10^{-04}	1.5×10^{-04}	3.2×10^{-02}	5.5×10^{-02}	1.2×10^{-02}	1.5×10^{-02}	2.3×10^{-04}	5.8×10^{-01}	9.1×10^{-02}	2.0×10^{-03}	4.4×10^{-01}	9.3×10^{-03}
	1 to 2 years	5.04×10^{-08}	1.26×10^{-09}	8.96×10^{-05}	2.67×10^{-03}	4.7×10^{-04}	1.0×10^{-01}	1.7×10^{-01}	3.7×10^{-02}	4.8×10^{-02}	7.2×10^{-04}	1.8×10^{00}	2.9×10^{-01}	6.4×10^{-03}	1.4×10^{00}	2.9×10^{-02}

	**4 to < 24 months**	**6.64×10^{-08}**	1.66×10^{-09}	**1.18×10^{-04}**	**3.52×10^{-03}**	**6.2×10^{-04}**	**1.3×10^{-01}**	**2.3×10^{-01}**	**4.9×10^{-02}**	**6.3×10^{-02}**	**9.4×10^{-04}**	**2.4×10^{00}**	**3.8×10^{-01}**	**8.4×10^{-03}**	**1.8×10^{00}**	**3.8×10^{-02}**
Rice (J)	0.5 to 1 year	1.60×10^{-08}	4.00×10^{-10}	2.43×10^{-03}	1.91×10^{-03}	1.00×10^{-04}	2.77×10^{-01}	3.75×10^{-01}	2.52×10^{-02}	8.61×10^{-03}	1.24×10^{-02}	3.72×10^{-01}	6.05×10^{-01}	3.9×10^{-03}	3.00×10^{-01}	2.28×10^{-02}
	1 to 2 years	5.04×10^{-08}	1.26×10^{-09}	7.64×10^{-03}	6.01×10^{-03}	3.15×10^{-04}	8.72×10^{-01}	1.18×10^{00}	7.94×10^{-02}	2.71×10^{-02}	3.91×10^{-02}	1.17×10^{00}	1.91×10^{00}	1.2×10^{-02}	9.47×10^{-01}	7.20×10^{-02}
	**4 to < 24 months**	**6.64×10^{-08}**	**1.66×10^{-09}**	**1.01×10^{-02}**	**7.92×10^{-03}**	**4.16×10^{-04}**	**1.15×10^{00}**	**1.55×10^{00}**	**1.05×10^{-01}**	**3.57×10^{-02}**	**5.15×10^{-02}**	**1.54×10^{00}**	**2.51×10^{00}**	**1.60×10^{-02}**	**1.25×10^{00}**	**9.48×10^{-02}**
Rice (K)	0.5 to 1 year	6.92×10^{-03}	4.00×10^{-10}	2.69×10^{-03}	3.73×10^{-03}	7.62×10^{-05}	3.29×10^{-01}	5.50×10^{-01}	3.21×10^{-02}	3.20×10^{-01}	8.64×10^{-05}	6.48×10^{-01}	6.06×10^{-01}	8.00E-11	2.49×10^{00}	1.13×10^{-02}
	1 to 2 years	2.18×10^{-02}	1.26×10^{-09}	8.48×10^{-03}	1.18×10^{-02}	2.40×10^{-04}	1.03×10^{00}	1.73×10^{00}	1.01×10^{-01}	1.01×10^{00}	2.72×10^{-04}	2.04×10^{00}	1.91×10^{00}	2.52×10^{-10}	7.85×10^{00}	3.56×10^{-02}
	**4 to < 24 months**	**2.87×10^{-02}**	**1.66×10^{-09}**	**1.12×10^{-02}**	**1.55×10^{-02}**	**3.16×10^{-04}**	**1.36×10^{00}**	**2.28×10^{00}**	**1.33×10^{-01}**	**1.33×10^{00}**	**3.59×10^{-04}**	**2.69×10^{00}**	**2.52×10^{00}**	**3.32×10^{-10}**	**1.03×10^{01}**	**4.69×10^{-02}**
Rice (L)	0.5 to 1 year	1.60×10^{-08}	4.00×10^{-10}	1.72×10^{-03}	4.45×10^{-03}	6.44×10^{-05}	3.52×10^{-01}	6.13×10^{-01}	2.50×10^{-02}	1.05×10^{-02}	2.07×10^{-03}	6.77×10^{-01}	6.70×10^{-01}	8.83×10^{-04}	2.78×10^{00}	1.46×10^{-02}
	1 to 2 years	5.04×10^{-08}	1.26×10^{-09}	5.40×10^{-03}	1.40×10^{-02}	2.03×10^{-04}	1.11×10^{00}	1.93×10^{00}	7.88×10^{-02}	3.31×10^{-02}	6.51×10^{-03}	2.13×10^{00}	2.11×10^{00}	2.78×10^{-03}	8.77×10^{00}	4.61×10^{-02}
	**4 to < 24 months**	**6.64×10^{-08}**	**1.66×10^{-09}**	**7.12×10^{-03}**	**1.85×10^{-02}**	**2.67×10^{-04}**	**1.46×10^{00}**	**2.54×10^{00}**	**1.04×10^{-01}**	**4.36×10^{-02}**	**8.58×10^{-03}**	**2.81×10^{00}**	**2.78×10^{00}**	**3.67×10^{-03}**	**1.16×10^{01}**	**6.08×10^{-02}**
Rice (M)	0.5 to 1 year	1.05×10^{-02}	2.48×10^{-03}	4.54×10^{-03}	2.82×10^{-03}	2.84×10^{-04}	3.02×10^{-01}	7.52×10^{-01}	2.67×10^{-02}	8.99×10^{-03}	3.01×10^{-03}	6.64×10^{-01}	7.80×10^{-01}	1.30×10^{-04}	3.22×10^{00}	9.38×10^{-03}
	1 to 2 years	3.32×10^{-02}	7.83×10^{-03}	1.43×10^{-02}	8.90×10^{-03}	8.96×10^{-04}	9.52×10^{-01}	2.37×10^{00}	8.40×10^{-02}	2.83×10^{-02}	9.48×10^{-03}	2.09×10^{00}	2.46×10^{00}	4.10×10^{-04}	1.01×10^{01}	2.95×10^{-02}
	**4 to < 24 months**	**4.37×10^{-02}**	**1.03×10^{-02}**	**1.88×10^{-02}**	**1.17×10^{-02}**	**1.18×10^{-03}**	**1.25×10^{00}**	**3.12×10^{00}**	**1.11×10^{-01}**	**3.73×10^{-02}**	**1.25×10^{-02}**	**2.75×10^{00}**	**3.24×10^{00}**	**5.40×10^{-04}**	**1.34×10^{01}**	**3.89×10^{-02}**
Rice (N)	0.5 to 1 year	8.77×10^{-03}	8.50×10^{-03}	1.18×10^{-03}	3.93×10^{-03}	7.46×10^{-05}	3.13×10^{-01}	4.84×10^{-01}	1.64×10^{-02}	1.53×10^{-02}	3.29×10^{-04}	6.12×10^{-01}	5.46×10^{-01}	8.00E-11	2.02×10^{00}	1.04×10^{-02}
	1 to 2 years	2.76×10^{-02}	2.68×10^{-02}	3.73×10^{-03}	1.24×10^{-02}	2.35×10^{-04}	9.85×10^{-01}	1.53×10^{00}	5.17×10^{-02}	4.82×10^{-02}	1.04×10^{-03}	1.93×10^{00}	1.72×10^{00}	2.52×10^{-10}	6.36×10^{00}	3.26×10^{-02}
	**4 to < 24 months**	**3.64×10^{-02}**	**3.53×10^{-02}**	**4.91×10^{-03}**	**1.63×10^{-02}**	**3.10×10^{-04}**	**1.30×10^{00}**	**2.01×10^{00}**	**6.81×10^{-02}**	**6.35×10^{-02}**	**1.36×10^{-03}**	**2.54×10^{00}**	**2.27×10^{00}**	**3.32×10^{-10}**	**8.37×10^{00}**	**4.30×10^{-02}**
Rice (O)	0.5 to 1 year	2.07×10^{-02}	4.00×10^{-10}	3.38×10^{-03}	1.20×10^{-02}	1.92×10^{-04}	4.69×10^{-01}	8.07×10^{-01}	3.85×10^{-02}	1.71×10^{-02}	4.73×10^{-04}	5.70×10^{-01}	8.96×10^{-01}	1.38×10^{-03}	2.72×10^{00}	7.12×10^{-03}
	1 to 2 years	6.51×10^{-02}	1.26×10^{-09}	1.07×10^{-02}	3.77×10^{-02}	6.06×10^{-04}	1.48×10^{00}	2.54×10^{00}	1.21×10^{-01}	5.39×10^{-02}	1.49×10^{-03}	1.80×10^{00}	2.82×10^{00}	4.36×10^{-03}	8.57×10^{00}	2.24×10^{-02}
	**4 to < 24 months**	**8.58×10^{-02}**	**1.66×10^{-09}**	**1.40×10^{-02}**	**4.96×10^{-02}**	**7.98×10^{-04}**	**1.94×10^{00}**	**3.35×10^{00}**	**1.60×10^{-01}**	**7.10×10^{-02}**	**1.96×10^{-03}**	**2.37×10^{00}**	**3.72×10^{00}**	**5.75×10^{-03}**	**1.13×10^{01}**	**2.96×10^{-02}**
Rice (P)	0.5 to 1 year	5.44×10^{-03}	1.26×10^{-02}	9.54×10^{-03}	8.73×10^{-03}	2.26×10^{-04}	6.88×10^{-01}	7.69×10^{-01}	6.30×10^{-02}	1.37×10^{-02}	1.06×10^{-02}	3.08×10^{-01}	4.62×10^{-01}	7.94×10^{-03}	1.58×10^{00}	7.48×10^{-03}
	1 to 2 years	1.71×10^{-02}	3.96×10^{-02}	3.00×10^{-02}	2.75×10^{-02}	7.11×10^{-04}	2.17×10^{00}	2.42×10^{00}	1.98×10^{-01}	4.30×10^{-02}	3.33×10^{-02}	9.70×10^{-01}	1.46×10^{00}	2.50×10^{-02}	4.97×10^{00}	2.36×10^{-02}
	**4 to < 24 months**	**2.26×10^{-02}**	**5.22×10^{-02}**	**3.96×10^{-02}**	**3.62×10^{-02}**	**9.37×10^{-04}**	**2.85×10^{00}**	**3.19×10^{00}**	**2.61×10^{-01}**	**5.67×10^{-02}**	**4.38×10^{-02}**	**1.28×10^{00}**	**1.92×10^{00}**	**3.29×10^{-02}**	**6.55×10^{00}**	**3.11×10^{-02}**
Rice (Q)	0.5 to 1 year	1.55×10^{-02}	4.00×10^{-10}	3.53×10^{-03}	5.31×10^{-03}	4.09×10^{-05}	4.59×10^{-01}	5.31×10^{-01}	3.13×10^{-02}	4.33×10^{-03}	1.85×10^{-02}	2.85×10^{-01}	6.98×10^{-01}	5.68×10^{-03}	1.82×10^{00}	1.52×10^{-02}
	1 to 2 years	4.90×10^{-02}	1.26×10^{-09}	1.11×10^{-02}	1.67×10^{-02}	1.29×10^{-04}	1.44×10^{00}	1.67×10^{00}	9.85×10^{-02}	1.36×10^{-02}	5.82×10^{-02}	8.98×10^{-01}	2.20×10^{00}	1.79×10^{-02}	5.74×10^{00}	4.78×10^{-02}
	**4 to < 24 months**	**6.45×10^{-02}**	**1.66×10^{-09}**	**1.47×10^{-02}**	**2.20×10^{-02}**	**1.70×10^{-04}**	**1.90×10^{00}**	**2.20×10^{00}**	**1.30×10^{-01}**	**1.80×10^{-02}**	**7.66×10^{-02}**	**1.18×10^{00}**	**2.90×10^{00}**	**2.36×10^{-02}**	**7.56×10^{00}**	**6.30×10^{-02}**
Rice (R)	0.5 to 1 year	3.50×10^{-04}	4.00×10^{-10}	4.50×10^{-04}	2.37×10^{-03}	2.25×10^{-04}	5.36×10^{-02}	8.62×10^{-02}	1.13×10^{-02}	2.65×10^{-01}	1.39×10^{-05}	6.94×10^{-01}	8.15×10^{-02}	8.00E-11	3.34×10^{-01}	1.43×10^{-02}
	1 to 2 years	1.10×10^{-03}	1.26×10^{-09}	1.42×10^{-03}	7.46×10^{-03}	7.09×10^{-04}	1.69×10^{-01}	2.72×10^{-01}	3.57×10^{-02}	8.34×10^{-01}	4.38×10^{-05}	2.19×10^{00}	2.57×10^{-01}	2.52×10^{-10}	1.05×10^{00}	4.50×10^{-02}
	**4 to < 24 months**	**1.45×10^{-03}**	**1.66×10^{-09}**	**1.87×10^{-03}**	**9.83×10^{-03}**	**9.35×10^{-04}**	**2.23×10^{-01}**	**3.58×10^{-01}**	**4.71×10^{-02}**	**1.10×10^{00}**	**5.77×10^{-05}**	**2.88×10^{00}**	**3.38×10^{-01}**	**3.32×10^{-10}**	**1.38×10^{00}**	**5.93×10^{-02}**

Ag = silver; Al = aluminum; As = arsenic; B = boron; Ba = barium; Cd = cadmium; Co = cobalt; Cr = chromium; Cu = copper; Mn = manganese; Ni = nickel; Pb = lead; Se = selenium; Sr = strontium; Zn = zinc..
*Shading indicates hazard quotients > 1.

Table S5

Estimated hazard quotients* for exposure to inorganic arsenic, if it is assumed to account for 52% or 100% of the total arsenic.

Type of infant cereal (ID)	HQ	
	iAs (52%)	iAs (100%)
Corn (A)	5.47×10^{-02}	1.05×10^{-01}
Oatmeal (B)	1.44×10^{-01}	2.77×10^{-01}
Oatmeal (C)	7.24×10^{-02}	1.39×10^{-01}
Multi-grain (D)	1.48×10^{-01}	2.85×10^{-01}
Rice and oat (E)	3.03×10^{-01}	5.83×10^{-01}
Rice and oat (F)	1.41×10^{00}	2.72×10^{00}
Rice and fruit (G)	1.05×10^{00}	2.01×10^{00}
Rice and cornstarch (H)	2.18×10^{-01}	4.19×10^{-01}
Rice and cornstarch (I)	1.96×10^{-01}	3.77×10^{-01}
Rice (J)	1.31×10^{00}	2.51×10^{00}
Rice (K)	1.31×10^{00}	2.52×10^{00}
Rice (L)	1.45×10^{00}	2.78×10^{00}
Rice (M)	1.68×10^{00}	3.24×10^{00}
Rice (N)	1.18×10^{00}	2.27×10^{00}
Rice (O)	1.93×10^{00}	3.72×10^{00}
Rice (P)	9.98×10^{-01}	1.92×10^{00}
Rice (Q)	1.51×10^{00}	2.90×10^{00}
Rice (R)	1.76×10^{-01}	3.38×10^{-01}

iAs = inorganic arsenic

HQ = hazard quotient; iAs = inorganic arsenic.

*Shading indicates hazard quotients > 1.
